# The state of the art and future trends of root canal files from the perspective of patent analysis: a study design

**DOI:** 10.1186/s12938-022-01060-0

**Published:** 2022-12-24

**Authors:** Jingang Jiang, Jianpeng Sun, Zhiyuan Huang, Zhuming Bi, Guang Yu, Jingwen Yang, Yong Wang

**Affiliations:** 1 Key Laboratory of Advanced Manufacturing and Intelligent Technology, Ministry of Education, Harbin University of Science and Technology, Harbin, 150080 Heilongjiang People’s Republic of China; 2 State Key Laboratory of Robotics and System, Harbin Institute of Technology, Harbin, 150001 Heilongjiang People’s Republic of China; 3 Department of Civil and Mechanical Engineering, Purdue University Fort Wayne, West Lafayette, 46805 USA; 4 Department of Mechanical Engineering, Tsinghua University, Beijing, 100084 People’s Republic of China; 5 National Engineering Laboratory for Digital and Material Technology of Stomatology, Peking University School of Stomatology, Beijing, 100081 People’s Republic of China; 6 Peking University School of Stomatology, Beijing, 100081 People’s Republic of China

**Keywords:** Dental materials, Dental instruments, Patent analysis, Root canal therapy, Root canal file

## Abstract

The goal of this review is to present a detailed and comprehensive description of the published work from the past decade regarding methods of improved material, geometric design, and additional functions in root canal files. The main improved methods of files and the most common technologies were further addressed, underlining their advantages and main limitations. Online databases (the Derwent Innovations Index) were consulted on this topic. Published work from 2010 to 2022 was collected and analyzed the relevant papers were chosen for inclusion in this review. The patent map classified the latest phase of the root canal files based on the analysis of the number of patents. The performance of the root canal files, such as materials. Directly affects the quality of the root canal therapy. We provided a thorough review of advances in the field of root canal files. In particular, three categories of improved methods were examined and compared, including material-based methods, geometry-based methods, and those based on additional functions. To understand this state of the art of different improved methods of root canal files, we conducted a literature analysis and a series of comparisons between different methods. The features and limitations of each method of root canal files were further discussed. Finally, we identified promising research directions in advancing the methods for the improved performance of root canal files. There is no perfect technology for all material/geometric design/additional functions, capable alone of fulfilling all the specificity and necessities of every patient. Although it is very promising, the material of the files remains understudied, and further work is required to make material science a pervasive technology in root canal therapy, and contribute to endodontic and periapical diseases by assisting in the subsequent development of root canal files.

## Background

Endodontic and periapical diseases are the more common pathologies in dentistry [1, 2]. The cause of the disease is an invasion of bacteria into the pulp through periodontal or defective areas of teeth, causing infection, or through physical channels, causing pain, bleeding, or even necrosis of the pulp [3]. Currently, the most effective treatment for endodontic and periapical diseases is root canal therapy [4]. Root canal therapy, also known as endodontic treatment, is a procedure in dentistry to treat necrotic pulp and root infections. The process of root canal therapy, i.e., mechanical preparation and chemical flushing to remove most of the infected material from the root canal [5, 6], followed by root canal filling and crown sealing [7]. The aim is to prevent the occurrence of periapical disease or to promote the healing of periapical disease that has already occurred [8]. Theoretically, the success rate of root canal therapy is between 83% and 97.1% [9]. However, the success rate of endodontic treatment is lower than the theoretical success rate, as shown in the surveys of the past decades [10]. The structural complexity of root canals, unskillful preparation technique, and insufficient performance of root canal files are all factors that contribute to the low success rate of root canal therapy in practice [11]. The solution to the first two problems will face great difficulties: for one, the root canal of a tooth is complex and variable, with a multidimensional curvature [12, 13], as well as many finer branches [14], which are inherent factors and cannot be changed artificially. Secondly, the current imbalance in the doctor–patient ratio in dentistry requires reliance on the clinician's manual operation combined with extensive clinical experience, so it is difficult to improve preparatory technology in a short period. Therefore, to solve the current problem of low success rate, improvement of root canal file performance is a key aspect.

**Fig.1:**
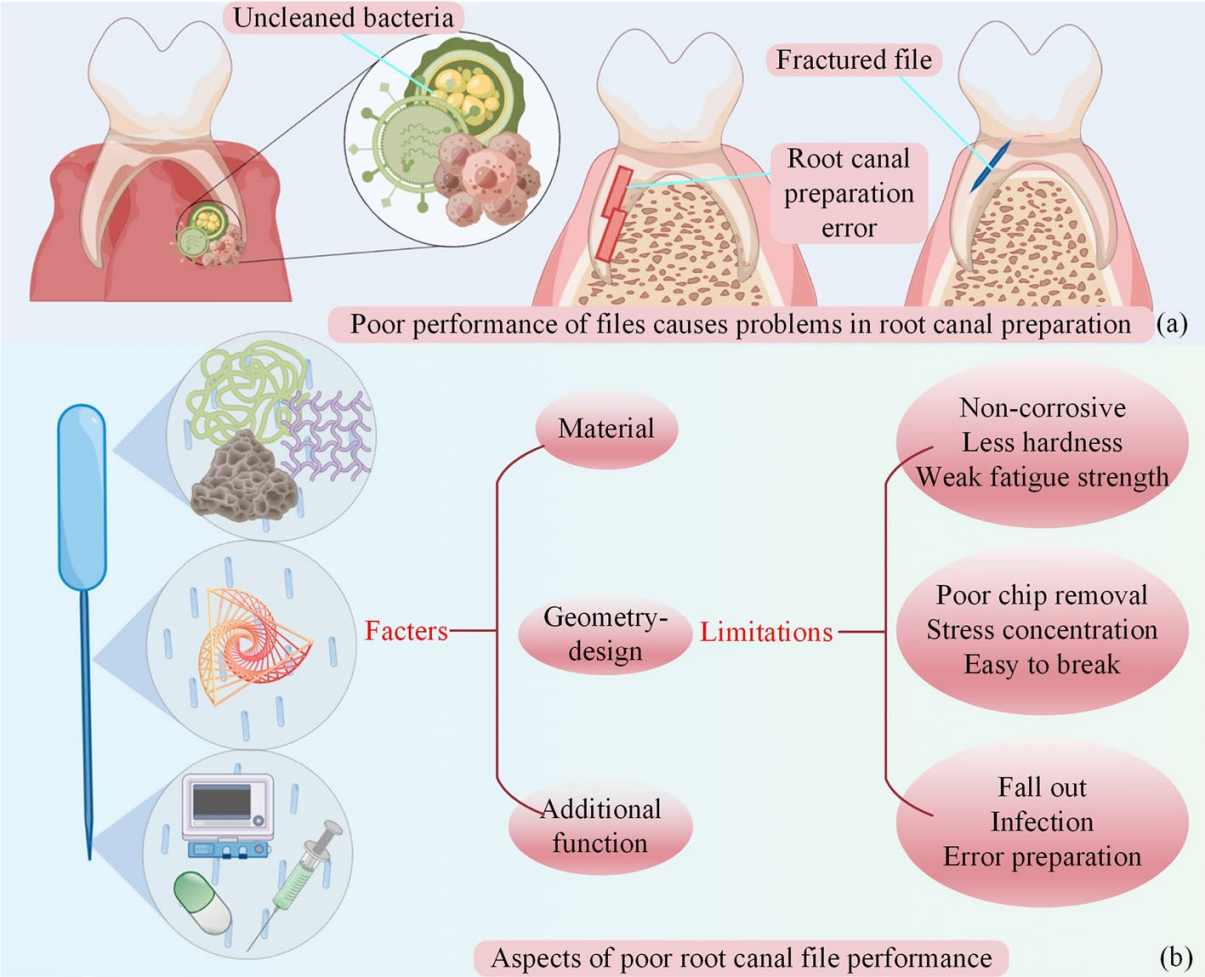
(Draw by Figdraw). Introduction to root canal therapy.** a** Problems of root canal therapy.** b** Problems with files

As an important tool in the mechanical preparation step, the quality of mechanical preparation is directly influenced by the performance of root canal files, which should create a regular and smooth tapered structure to avoid significant deviation from the original shape and orientation of the canal. The quality of mechanical preparation directly affects the success rate of root canal therapy. However, from the clinical use, the performance of root canal files does not meet the requirements of required mechanical preparation. As shown in Fig. 1(a), the poor performance of root canal files can cause several problems, such as apical inflammation, incorrect preparation, and instrument separation.

This paper introduced the main solution to these problems is to improve the performance of root canal files. The properties of files can be divided into mechanical properties and use properties. The deficiency of mechanical properties is reflected in material limitations and poor geometric design, and the deficiency of use properties is reflected in a single function. As shown in Fig. 1(b), the factors affecting the performance of root canal files and their current limitations were described. For dental medical devices [15–18] in dentistry, an extensive review of literature exists but a detailed overview of root canal files is, to the author’s best knowledge, missing.

The main motivation of this paper is to provide a comprehensive survey of improved methods of root canal files, focusing on the improved principles as well as improved characteristics of various methods, which are expected to improve therapeutic efficiency, reduce accident occurrence, lower costs, and eventually achieve the high-precision treatment of pulp disease and periapical disease, and occlusal relationship of patients can be reproduced.

The rest of the paper is organized as follows. In "Material" Section, this review proposed the methods of literature analysis and structure of this paper. Secondly, the root canal files were divided into materials, geometry, and functions, which are in "Additional function" Section. In "Discussion" Section and "Future trends" Section, the current status of root canal files and their future directions were discussed. Finally, the full paper is summarized in "Conclusion" Section.

### Root canal files patent data acquisition

Mathematical and statistical methods were used in bibliometrics to quantitatively describe and evaluate various external characteristics of scientific literature to understand the state of research and predict trends in scientific and technological development. In this paper, Patentics [19, 20] was selected as the literature analysis tool. With the world's original Patentics intelligent semantic mathematical model, only by inputting a key technology point, the technology lineage and technology route related to the technology can be automatically analyzed by correlation clustering, and the high-precision correlation relationship from hyperspace can be projected to a 2D map space through the mapping of correlation retention. It can retrieve and download full text of patents of the US, Japan, China, Europe, and WIPO(PCT), and make patent analyses on retrieval results [21]. Patentics is currently used in the inspection of complex devices, such as chemical devices [22], circuit design devices [23], and biological devices [24]. Since a root canal file is just a little medical device, it is difficult to identify suitable search elements and conduct a targeted search during the search process due to the complexity of its structure and the wide range of fields involved. Patentics intelligent semantic search has rich search fields, and with reasonable human intervention, it can improve the search efficiency of root canal upgrading methods, quickly browse the literature with novelty and creativity, and effectively improve the search efficiency. And as an important tool for treating endodontic disease, the effective retrieval of its elevation method is of great significance for treating endodontic and periapical diseases.

**Fig. 2:**
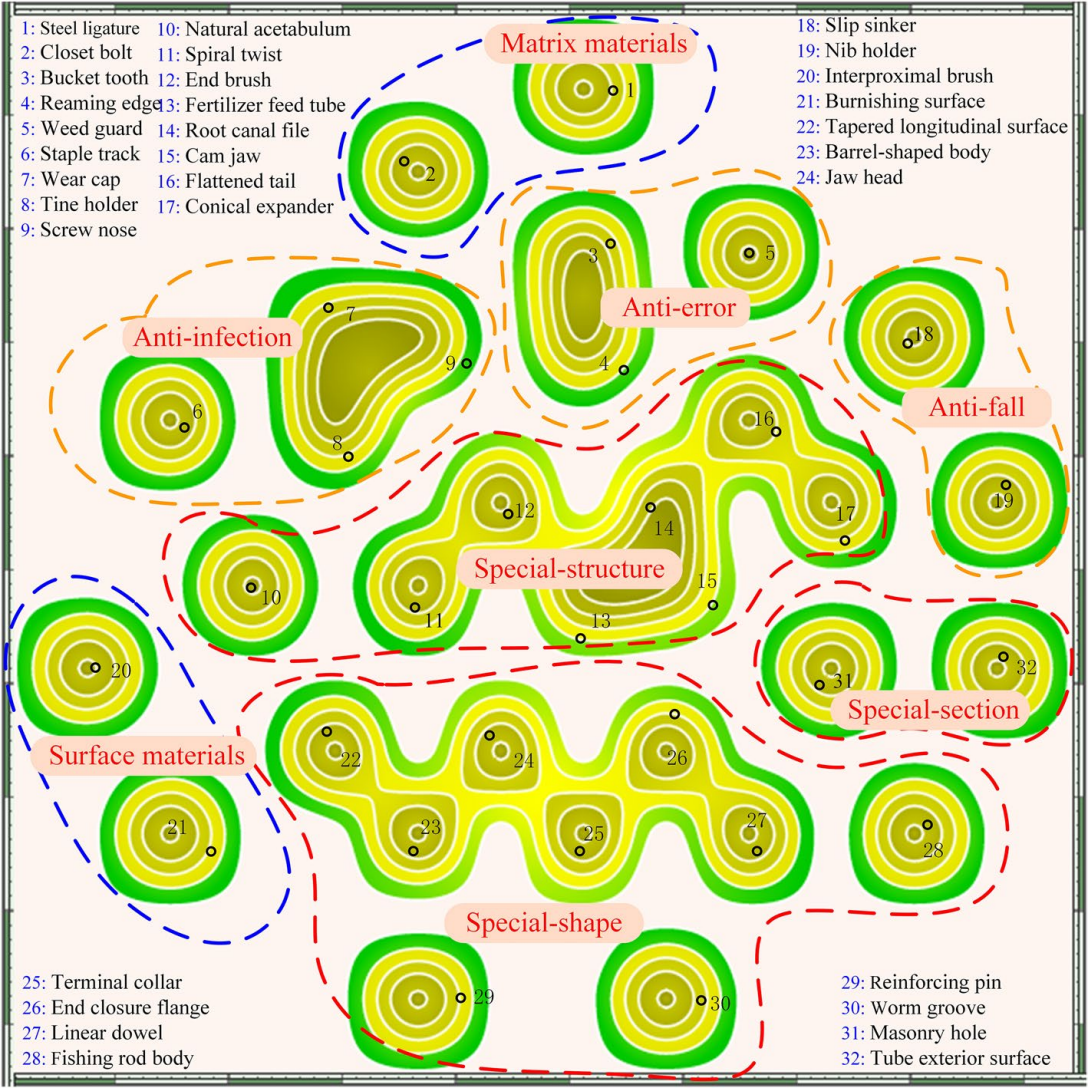
Patent map of root canal files

The Derwent Innovations Index was used as the main source of relevant literature by specifying the key technical point of the “root canal files,” the language of “English,” and the “core” collection of the Derwent Innovations Index. The corresponding search resulted in 626 documents from 1975 to 2022. The exported documents were analyzed by Patentics to determine current research hotspots and key technologies in the field of root canal files. The keywords in Patentics were selected for a co-occurrence network analysis. Based on the above mentioned, methods to improve the performance of root canal documentation can be deduced. As shown in Fig. 2, each keyword was taken as a node; there were 32 nodes in the co-occurrence network analysis. As shown by the nodes enclosed by dashed lines in the figure, the nodes within the dashed lines represent an enhancement method. The methods to improve the performance of root canal files were classified into 8 subcategories. The keywords represented by each node were marked in detail. If two keywords were used in one article, the corresponding nodes were met in the map. There were different colors to indicate different applicant rankings, and different positions indicate different applicant citations. The patent information was visualized as a contour topographic map to represent the peaks and valleys of patent distribution. The closer to the top of the mountain indicates that the keyword is hotter. The meaning of the same contour represents the proximity of the number of patents. The technology lineage and technology route related to the root canal files are shown in Fig. 2. According to the clustering results of the patent map, it can be found that the methods to improve the performance of root canal files mainly focus on three major methods: material, geometry, and additional function. The search strategy identified 626 potentially relevant records, and 445 remained after duplicate removal. Root canal therapy failure is characterized by the complexity of the root canal structure. Manual root canal files are often used in the preparation of such canals because mechanical root canal files are effective but difficult to control. In this paper, the focus is on manual root canal files because the goal is to improve the success rate of root canal therapy. After screening for title and timing, 87 studies from 2010 to 2022 were assessed as eligible.

**Fig. 3:**
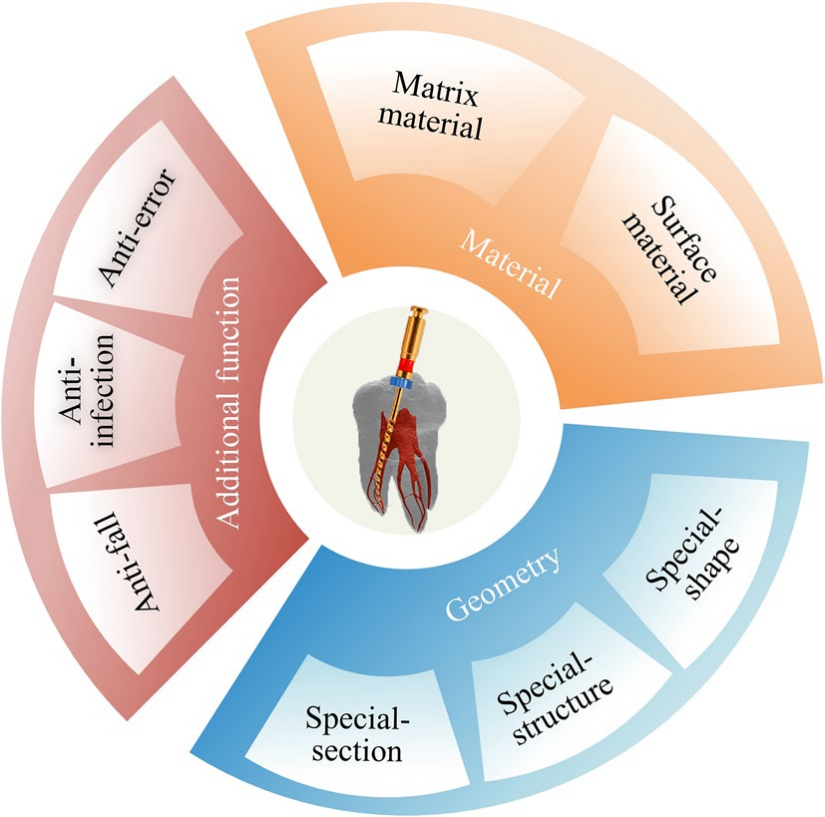
Classification of root canal files according to performance

So, in this paper, the root canal files of the last decade were classified into three major categories based on performance improvement methods: material based, geometry based, and those base on additional functions. Moreover, the methods of material based were divided into matrix material methods and surface material methods. The geometry-based methods were divided into special-shape methods, special-structure methods, and special-section methods. Additional function methods were divided into the anti-fall methods, anti-infection, and anti-error methods as shown in Fig. 3.

### Classification of root canal files

#### Material

Statistical studies [25] have confirmed that the main reason for root canal therapy failure is the fragile performance of the instruments, which is the most common problem in the clinical use. But the probability of instrument fracture can be reduced by optimizing the mechanical properties of root canal files. Improvements in material properties, manufacturing process, and geometric design can improve the mechanical properties of root canal files, which are directly influenced by material properties. The common materials used for root canal therapy instruments are stainless-steel and nickel-titanium alloy, and the traditional stainless-steel root canal files have good cutting properties due to their high strength, so they can easily cause steps and lateral penetration on the inner surface of the canal [26]. Nickel-titanium has gradually replaced stainless-steel instruments as the tool of choice for root canal therapy [27] because of its significant advantages in terms of flexibility, formability, memory properties, and efficiency. However, in clinical use, nickel-titanium root canal files may break and separate due to material failure during multiple uses or when complex root canals are encountered [28]. For the problem of material failure, according to the different methods of improving material performance, this section has introduced the methods of improving materials into matrix material and surface material.

#### Matrix material

Nickel-titanium alloys were first used in the manufacture of root canal files by Walia [29] in 1988. With its good biocompatibility and damping properties, NiTi alloy can significantly improve the elasticity and fracture resistance of root canal files and reduce complications during root canal therapy. It is now widely used in clinical use [30]. However, when a nickel-titanium root canal file is used to prepare a bent root canal, it may break due to torsional fatigue or bending cycle fatigue, which can seriously affect the completion of the root canal therapy. Therefore, how to reduce fracture is the focus of clinical research [31]. Scholars have conducted numerous studies on the matrix materials intending to improve the fatigue resistance and flexibility of root canal files. These methods can be better adapted to the root canal pattern and reduce the risk of instrument separation during root canal treatment.

**Fig. 4:**
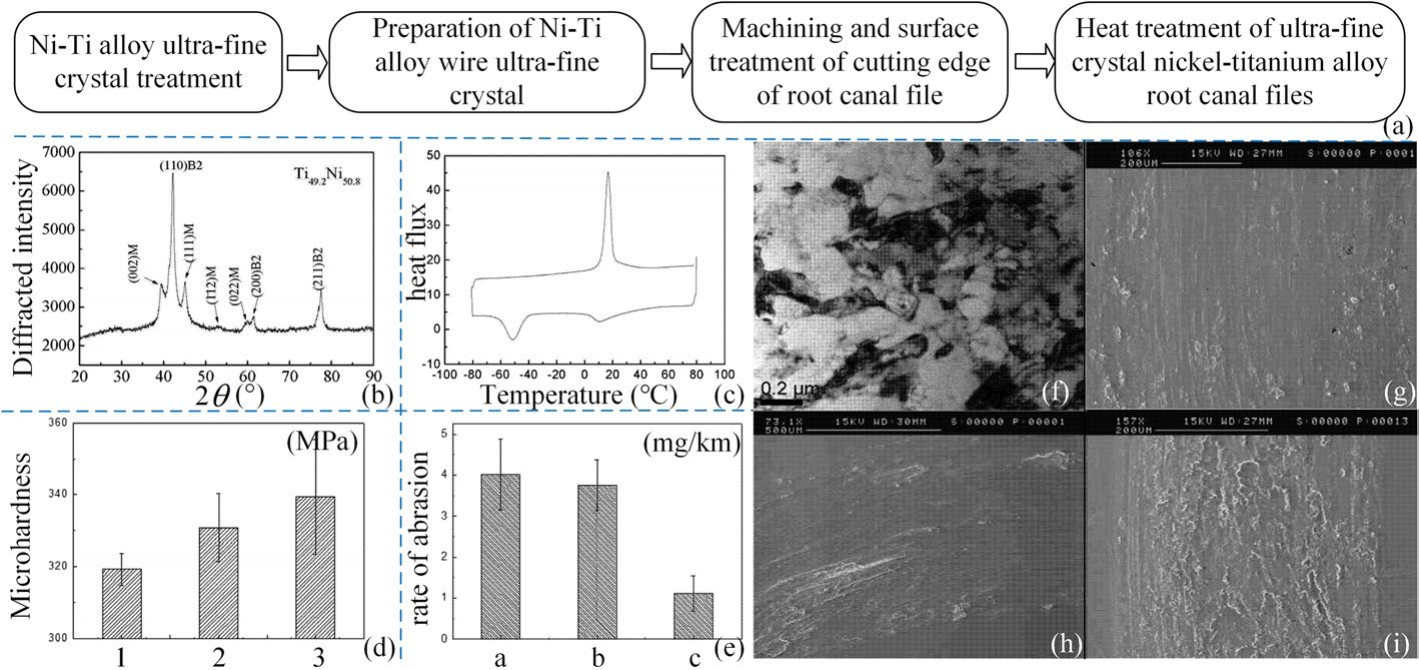
Preparation of ultrafine-grained nickel-titanium alloy root canal files. Reproduced with permission. Source: CNIPA, www.cnipa.gov.cn. **a** The preparation process of ultrafine-grained nickel-titanium alloy root canal files. **b** XRD graph. **c** DSC curves. **d** Microhardness plots of nickel-titanium, superfine-grained nickel-titanium, and superfine-grained nickel-titanium alloys after heat treatment at 400 °C. Figures 1, 2, and 3 indicate the microhardness plots of nickel-titanium alloy and ultrafine-grain nickel-titanium alloy, and 3 indicates the microhardness plots of ultrafine-grain nickel-titanium alloy after heat treatment at 400 °C. **e** Wear rate diagrams of nickel-titanium alloy, ultrafine-grain nickel-titanium alloy, and ultrafine product nickel-titanium alloy after heat treatment at 400 °C. Figures a and b indicate the wear rate graphs of nickel-titanium alloy and ultrafine-grain nickel-titanium alloy, and c indicates the wear rate graph of ultrafine-grain nickel-titanium alloy after heat treatment at 400 °C. **f** Electron micrographs of the prepared ultrafine-grain nickel-titanium alloy root canal files. **g** Wear surface morphology of nickel-titanium alloy. **h** Wear surface morphology of ultrafine-grain nickel-titanium alloy. **i** Wear surface morphology of ultrafine-grain nickel-titanium alloy after heat treatment at 400 °C

A gradient flexible nickel-titanium root canal files [32] was proposed by Wang Z. The files have excellent cutting properties, but the file material is not set for the preparation requirements of the root canal. Later, he improved the files according to the demand for material [33] in the different part of canal. The internal organization of the tip part of the root canal files was improved to the martensitic M phase, the middle part to the R phase, and the root part to the austenitic A phase. After this method of manufacturing, it can effectively prevent side penetration. This treatment solves the problem of hardness and wear resistance of existing nickel-titanium root canal files. Zheng YF proposed ultrafine-grain nickel-titanium alloy root canal files [34], and the preparation process is shown in Fig. 4(a). The material composition of the files is martensitic and austenitic when not in use, and austenitic in clinical use. From Fig. 4(d, e), it can be seen that the hardness and wear resistance of the superfine grain-treated nickel-titanium alloy and the superfine grain nickel-titanium alloy after heat treatment by holding at 400 °C for 60 min are improved [34]. As shown in Fig. 4(g–i), nickel-titanium and superfine grain nickel-titanium alloys are dominated by abrasive wear, while superfine grain nickel-titanium alloys after heat treatment are dominated by adhesive wear.

In order to solve the problem of large wobble at the end of the root canal file, many methods have been proposed by many scholars. Liu S proposed austenitic nickel-titanium alloy as the material for the connecting rod [35], and the threads at the edge are machined from a martensitic nickel-titanium alloy. This type of root canal file can effectively reduce the oscillation of the blade. With the development of minimally invasive dental techniques, the use of minimal-size root canal files is required. However, the conventional material of root canal files is not designed for the micro-preparation of root canals and is prone to fracture. In response to this problem, Steven S proposed polymeric materials [36]. It includes stainless steel, nickel-titanium, titanium, carbon steel, plastic, carbon fiber, or composite materials. In response to the problem that root canal files are not easily removed after accidental breakage, Duan JH proposed biodegradable magnesium alloy root canal files [37]. When a root canal file is broken, a corrosive degradation reaction occurs when the magnesium alloy at the section encounters the root canal flushing fluid. Because magnesium is a macronutrient in the body, it has good biocompatibility and is harmless to humans.

#### Surface material

There are many advantages in nickel-titanium alloys, but their disadvantages cannot be ignored, such as low surface hardness and poor corrosion resistance in root canal irrigation fluids. And in mechanical preparation, due to factors, such as wear, corrosion, and fatigue, resulting in micro-cracks on the metal surface of nickel-titanium files at the same time, disintegrating metal debris will be produced. The metal debris reacts with the tissue fluid and residual flushing fluid that exudes from the root canal, which in turn leaches out metal particles that are harmful to humans. Some reports show that nickel-chromium alloys will produce varying amounts of nickel ions after 7 days of immersion in artificial saliva. Due to the strong toxic side effects of heavy metal nickel ions on the human body, the release of nickel ions has become one of the indicators of biosafety evaluation of medical devices containing nickel metal [38]. In recent years, scholars have been working on surface modification techniques to improve the defects and deficiencies of root canal files in terms of biosafety, corrosion resistance, and fatigue fracture resistance [38–41]. For the surface modification of root canal files, the main modalities include polishing [42–45], coating metallic materials [53, 54], coating metals by magnetron sputtering process [56], and transition coating composite film [55].

Scholars have enhanced the properties of file surfaces by electrolytic polishing [42, 43], mechanical polishing [44], and chemical polishing [45]. Mechanical polishing can reduce the mechanical processing marks on the root canal files' surface and make the surface smoother, but the modification effect is not as significant as electrolytic polishing and chemical polishing. Electrolytic polishing and chemical polishing perform well in improving corrosion resistance and biocompatibility but are still affected by temperature and pH in the oral environment, and nickel ions can still precipitate from the surface of nickel-titanium root canal files and cause harm to humans [46]. To address this problem, surface coating technology can be an effective solution, which is more suitable for the surface modification of root canal files and can be achieved by coating the surface of nickel-titanium root canal files with a single film layer or a composite film layer to improve the performance [47–50]. It has been demonstrated [51] that the surface properties of the files can be improved by coating the surface of the nickel-titanium root canal files with a metal film to improve their hardness, wear resistance, and inhibit the precipitation of nickel ions from the surface. Zhang J proposed a method for the preparation of metallic titanium nitride oxide composite film [52], which can effectively inhibit the outward diffusion of nickel ions. The hardness and corrosion resistance of the surface of the nickel-titanium root canal files is improved while maintaining the original elasticity of the files. Huang BM proposed to wrap the metal tube layer by layer on the outside of the mandrel [53]. There is a certain margin between the metal tubes, and then the mandrel and metal tubes are drawn and annealed in one piece, which can make the root tube files have the advantages of no unscrewing, no fracture, and no jamming. Tenney R proposed a selective coating with coating materials [54], including metallic or non-metallic, inorganic-like fullerene structures, or complexes containing such nanostructures. The files itself is made of a composite of selected shape memory and super elastic materials. The selective coating, a solid lubricant, is used as a permanent coating, thereby reducing friction between the nickel-titanium root canal files and the root canal wall without affecting the unique properties of the shape memory alloy. However, the bond strength between the coating and the substrate may not be high.

**Fig. 5:**
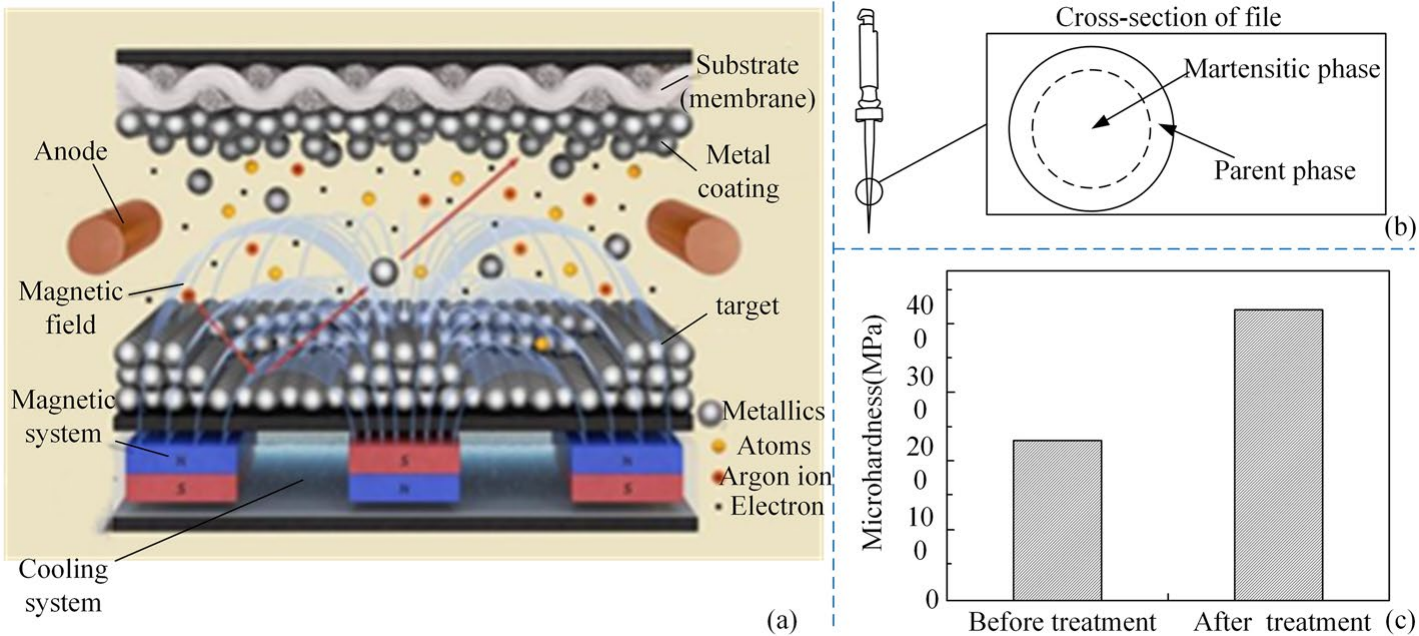
A surface modification method. Reproduced with permission. Source: CNIPA, www.cnipa.gov.cn. **a** The schematic diagram of the magnetron sputtering principle. **b** Schematic diagram of the cross-section of a nickel-titanium alloy root canal file. **c** Comparison of the hardness of nickel-titanium alloy root canal files after treatment and before treatment

For the problem of low bond strength between coating and matrix. Tong YX proposed the use of the magnetron sputtering process to deposit a metal coating on the surface of the root canal files [55]. Magnetron sputtering is the use of electric and orthogonal magnetic fields established on the target surface to control the targeted surface particles to be directed to the substrate surface, thus forming a coating on the substrate surface. The principle of magnetron sputtering is shown in Fig. 5(a, b) shows a schematic diagram of the cross-sectional material distribution of the root canal files after the above treatment. The hardness of the untreated and treated root canal files was tested using the HVS-100 digital display microhardness tester, and the results are shown in Fig. 5(c). It can be seen that the hardness of the root canal files is improved considerably, which means that the cutting efficiency is also improved. High-speed sputtering can substantially increase the deposition rate, but the low utilization of the target material is also a problem that needs to be solved in the future. Table 1 summarizes the machining process and treatment of material-based methods for root canal files.

**Table 1:** Overview of improved material methods of root canal files

Material-based	Material objects	Substrate internal materials/ Surface treatment materials	Processes/methods	Performance	Characteristics	Code
Matrix material	NiTi	MA and AU	CDA	Gradient flexible	GF	CN105852991 [32]
	NiTi	MA M phase MA R phase AU A phase	CDA	Adapt to canal	FR	CN107242911 [33]
	NiTi	MA and AU	HT, Ultrafine crystal process	HCE	WR	CN102743233 [34]
	NiTi	AU	Mechanical process	Different material application	GS	CN206518610 [35]
	Polymer	Stainless steel, NT	3D printing/mold making	Can be made smaller	GF, IS	WO2020243281 [36]
	Magnesium alloy	Magnesium and fluorine	Fluorine coating	Safety for the human body	Degradable, IS	CN203677273 [37]
Surface material	NiTi	Electrolytic materials	HT, CP	Improve surface performance	WR	US20110159458 [42]
	NiTi	High vacuum atmosphere compounding	Electrochemical polishing	Good glossy finish	FR	CN111685897 [43]
	NiTi	Polisher	Mechanical polishing	Save manufacturing time	Easy production	CN108788644 [44]
	NiTi	Chemistry	CP	Stabilize passivated oxide layer	FR, GF	US20160024311 [45]
	NiTi	TiZrON	Chemical composite membranes	Inhibits the diffusion of nickel ions	IS	CN105908136 [52]
	N/A	Metal	CDA, Reduced material process	Metal coating	GF	CN112453828 [53]
	NiTi	Fullerenes, NiTi, inorganic	Selective coating	Reduce the friction	IS	CN103096830 [54]
	NiTi	Metal	Magnetron Sputtering	High hardness	HCE	CN104630730 [55]

Materials/Processes: *NiTi* Nickel-titanium, *MA* Martensite, *AU* Austenite, *CDA* Cold drawn Annealed, *HT* Heat treatment, *CP* Chemical polishing

Performance/Characteristics: *GF* Good flexibility, *FR* Fatigue resistance, *WR* Wear resistance, *IS* Improve safety, *HCE* High cutting efficiency, *GS* Good stability

### Geometry

The potential mechanical properties are determined by the geometric design of the root canal file. However, it has been studied extensively because conventional root canal files cannot achieve efficiency and safety at the same time, such as improvements in the centerline [56–59], blade shape [63–66], spiral groove [67–71], tip diameter [61, 62], spatial structure [77–81], and cross-section [98–105] of the root canal files. It aims to enhance the potential mechanical properties of root canal files and optimize the mechanical properties and fatigue resistance.

#### Special-shape

Root canal files are flat in shape and are prone to jamming due to their low chip removal efficiency. Its handle structure also obstructs the clinicians' view and reduces the efficiency of preparation. The use of special-shape methods can solve this kind of problem.

**Fig. 6:**
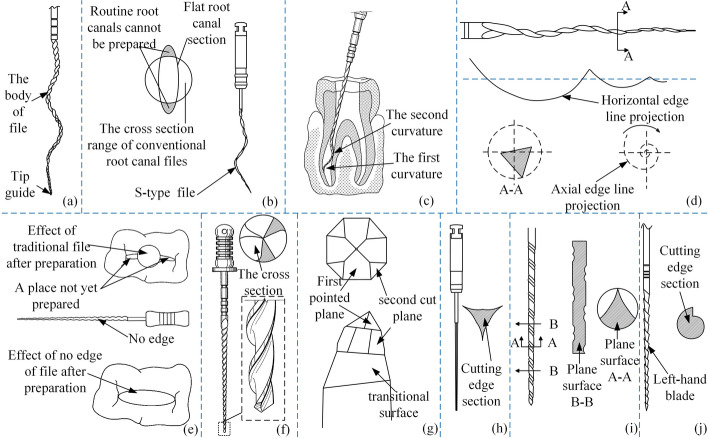
Irregular root canal files. Reproduced with permission. Source: USPTO, www.uspto.gov: CNIPA, www.cnipa.gov.cn. **a**–**b** 3D root canal files [56, 57]. **c** Multi-curvature root canal files [58]. **d** A spatial spiral shape [59]. **e** A root canal file with two edgeless sides [60]. **f** A guided tip structure [61]. **g** A first cusp and a second section at the tip of the file [62]. **h** A left-handed pattern [63]. **i** A vertical pattern [64]. **j** Increasing the chip-tolerant space [65]

Scholars have improved the root canal files' spatial structure to address the problem that the cutting edge does not fit closely to the root canal wall. For a better cut of root canal walls, Long XP and Long YF invented 3D root canal files [56, 57]. The structure is shown in Fig. 6(a, b). For cleaning a root canal in all directions, Johnson WB proposed multi-curvature root canal files [58] with at least two curvatures located in different planes. Figure 6(c) shows a schematic diagram of the files in operation. Wang Z proposed that the central axis of the working part of the root canal files should be designed as a spatial spiral shape [59], which is better adapted to the root canal's direction. The structure is schematically shown in Fig. 6(d). A root canal files with two edgeless sides has been proposed by Kou WZ to reduce the unnecessary cutting of dentin [60]. Figure 6(e) illustrates how a root canal can only be treated in one lateral or longitudinal direction. William J proposed a guided tip structure [61], as shown in Fig. 6(f), to reduce unnecessary resistance caused by the tip of the root canal files. Bai LL proposed a first cusp and a second section at the tip of the files [62] to improve guided travel and cleaning efficiency in root canals. The structure of the improved root canal files is shown in Fig. 6(g).

**Fig. 7:**
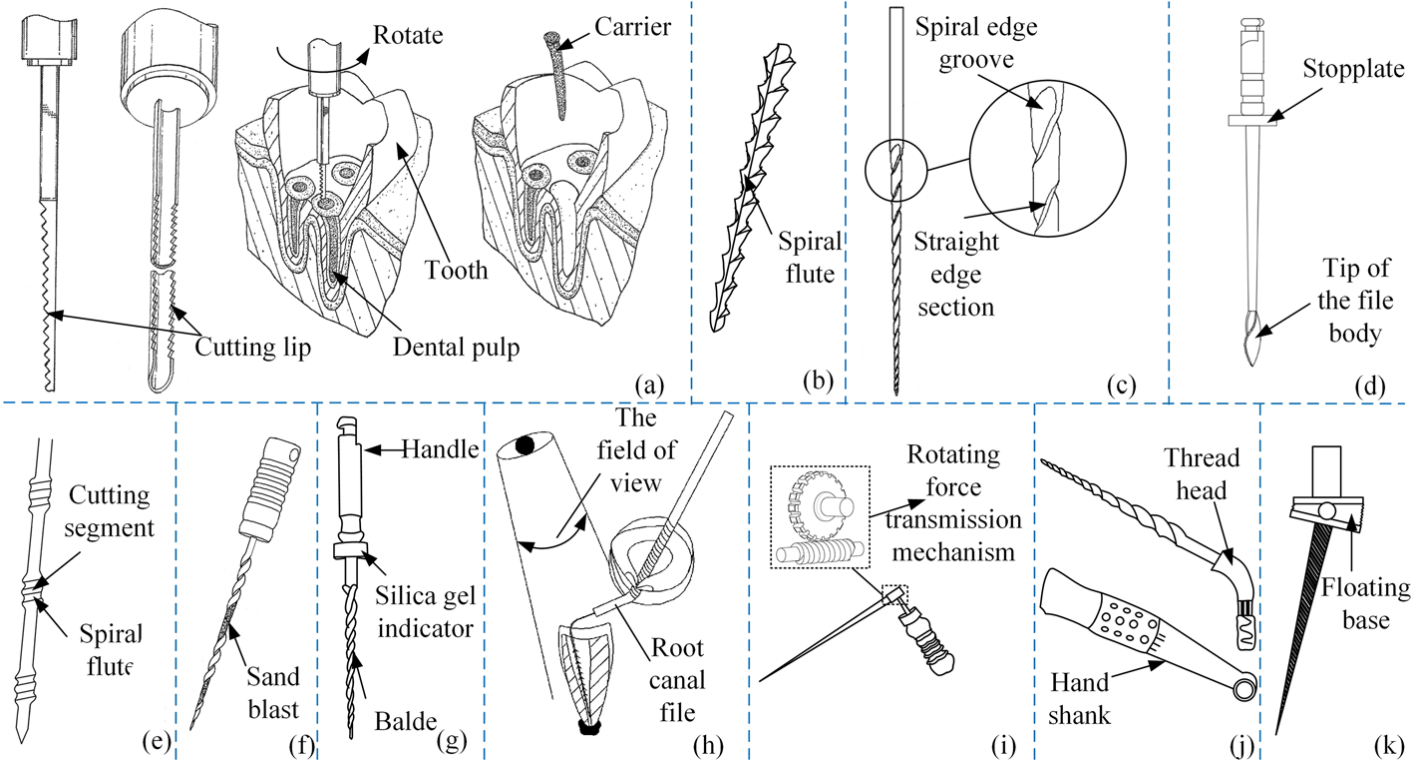
Discontinuous root canal files. Reproduced with permission. Source: USPTO, www.uspto.gov: CNIPA, www.cnipa.gov.cn. **a** A series of adjacent 3D pyramids [66]. **b**–**f** Discontinuous cutting edge [67–71]. **g**–**k** Root canal files for molar teeth [72–76]

About the root canal wall being embedded with the cutting edge, Long XP and Long YF changed the conventional cutting edge to a left-handed pattern [63] and a vertical pattern [64]. The structure is shown in Fig. 6(h, j). By increasing the chip-tolerant space, as shown in Fig. 6(i), Long XP avoided continuous embedding [65]. Farrag OAS proposed three types of files [66]. The first file’s cutting edge is side cutting with pyramidal teeth and ends in a pointed tip. The second type of file has a curved cross-section and a sharped tip, with pyramidal teeth lining the edges of the curved blade section. The third type of file has a flat debriding side that tapers to a point, with a series of adjacent 3D pyramids emerging from this surface for removing loose material or in urging already loosened material from a root canal, as shown in Fig. 7(a). Scholars also started from the continuity of cutting edge [67–71], which increased chip space and improved the chip removal function compared to the body of the conventional file. In this type of file, the edges are discontinuous, thus avoiding continuous embedding and making the solid part of the root canal files smaller. As shown in Fig. 7(b–f), it only plays an enlarging role during continuous cutting and can effectively prevent side penetration and the appearance of steps. Researchers have improved the handle structure [72–76] of root canal files in response to the problem of obstructing the line of sight when using root canal files, as shown in Fig. 7(g–k). The curved files make it easier for the dentist to treat patients with restricted openings or molar teeth, avoiding damage to other healthy areas. Table 2 summarizes the advantages and limitations of the special-shape methods for root canal files.

**Table 2:** Overview of special-shape methods of root canal files

Code	Special-shape	Performance	Advantage	Limitation
CN209595930 [56]	3D	SS	GCR	Insufficient strength
CN207400812 [57]	S-shape	SS	GCR	Weak chip removal function
US10136962 [58]	More curvature	SS	LR	No easy access to the inside of the root canal
CN209032679 [59]	Helical shape	SS	GC	Out of alignment with the root canal axis
CN201676027 [60]	Normal	Without two edges	LR	A single type of preparation
EP2140 828 [61]	Small tip	Three spiral grooves	LR	Small contact area affects cutting performance
CN211460595 [62]	Optimized tip	Two special surfaces	GCR	Non-streamline design tends to cause steps
CN203724240 [63]	Vertical cutting pattern	Vertical	NCE	Vertical grain affects chip removal efficiency
CN209933021 [64]	Multiple spiral grooves	Many	GCR	Too many spiral grooves affect the strength of the files
CN203943750 [65]	N/A	Left-hand blade	NCE	Left-handed blades are not versatile for users
US11083539 [66]	Tubular	Zigzag structure	NCE	Large size and inconvenient operation
CN109498186 [67]	Composite cutting edges	Discontinuous	NCE	The spiral groove is in the opposite direction of the cutting edge
CN213552491 [68]	Multi-pronged cone	Spiral	GCR	Not easy to control when rotating at a high speed
CN211884087 [69]	Smooth	None	IS	No cutting edge leads to poor cleaning ability
US20100119990 [70]	Interrupted shapes	Discontinuous	NCE	Continuously interrupting spiral grooves affect chip removal
EP3170471 [71]	Spiral wound wire sets	Discontinuous	NCE	Spiral wound wire sets may not be strong enough to bond with the substrate
CN206518609 [72]	Improved handle	Change to the universal handle	GV	Difficult to expand the scope of use
CN211049650 [73]	Improved handle	Extended handle	GV, GVI	The handle is too long to affect the operation
CN206910406 [74]	Improved handle and blade	Change of angle	GV	Unstable bending part
CN205339178 [75]	Improved handle	The changed direction of the handle	GV, GVI	Difficult to disassemble
CN108542513 [76]	Improved handle	Change to floating seat	GC, GV	Unstable force transmission during use

*GCR* Good chip removal, *SS* Space spiral, *LR* Low resistance, *GC* Good convenience, *NCE* No continuous embedding, *IS* Improve safety, *GV* Good versatility, *GVI* Good visibility

#### Special-structure

There are complex morphological structures and variants in the root canal. However, ordinary root canal files have a single range of preparation and are formed in one piece, making it difficult to clean the small roots within the root canal. In many cases, these tiny branches harbor bacteria and it is difficult to clean them with conventional root canal files. Consequently, many scholars proposed the composite structure method, which not only promotes full chemical action in the root canal but also better conforms to the root canal's shape.

**Fig.8:**
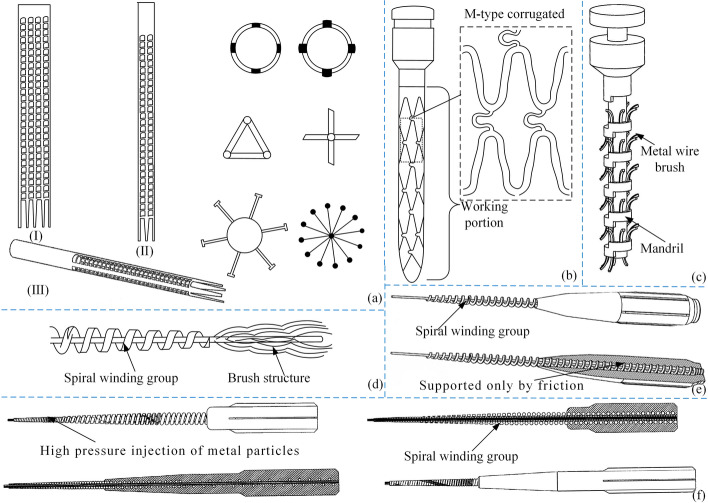
Self-adjusting root canal files. Reproduced with permission. Source: USPTO, www.uspto.gov: CNIPA, www.cnipa.gov.cn. **a**–**c** Use of variable profiles root canal files [77–81]. **d** Brush. **e**–**f** Spiral wound root canal files [82–84]

Figure 8(a–c) shows the structures [77–81] of the kinds of files. As the files' volume and use profiles change during use, the root canal is effectively cleaned without excessive widening of the root canal wall. Root canal walls are uniformly cut, which helps keep the root canal system in its original shape. More effective cleaning of flat, oval irregular root canals and root canal isthmus areas. The works of references [82–84] proposed to wind a spiral winding set on the surface of the file's body. According to the reference [82], when the files reach the root tip, rotation in the opposite direction creates a brush that sweeps debris out of the root canal. This is shown in Fig. 8(d). In reference [83], the elastomeric grip has an outer diameter that is slightly wider than an internal diameter of a barrel of a dental instrument whereby on insertion into the barrel it is supported therein only by friction. If the shear force between the files and the root canal increases beyond a predetermined value, the grip will slip in the handpiece barrel and no breakage damage will occur to the files, nor any undesirable damage to the dentine layer. The file structure is schematically shown in Fig. 8(e). In reference [84], besides a spiral winding around the body of the file, a high-pressure spray of metal particles is applied to its surface. A textured surface on the files is used to polish the walls of root canals. Figure 8(f) shows a cross-sectional view of the files in two embodiments in the vertical direction.

The composite structure makes it easier to carry the chemical irrigation agent into the root canal, which helps clean the necrotic debris and bacteria from the inner wall of the root canal and allows the solution to have the best dissolving properties on the tissue. The advantage of these root canal files is that they offer a greater cleaning range than conventional root canal files and better conform to root canal alignments, but the disadvantage is that they are not strong enough. Over-preparation may result in thinning of the root canal wall, but there is a risk of inadequate preparation and residual inflammatory material, leading to secondary recurrence.

**Fig. 9:**
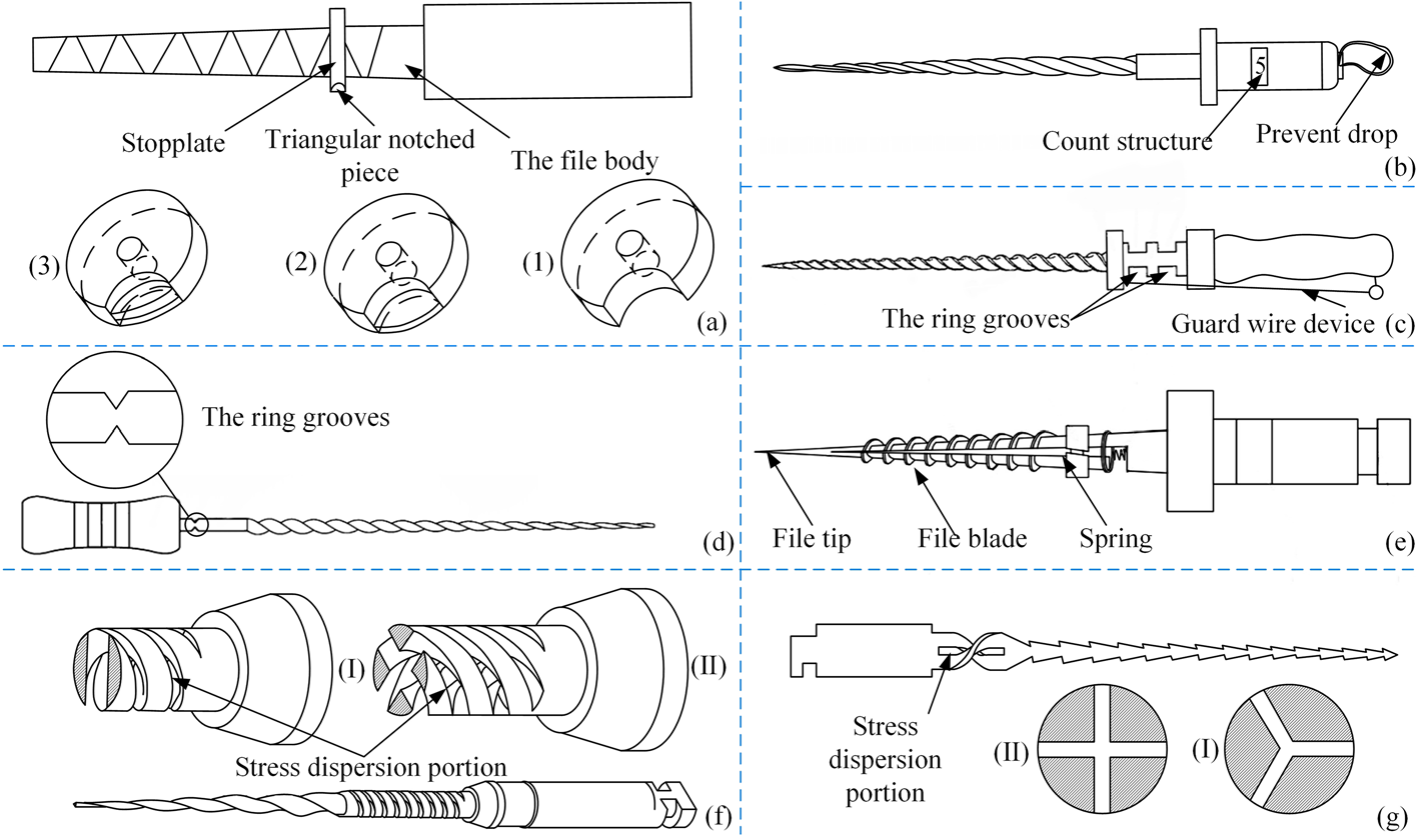
Prevent fracture of root canal files. Reproduced with permission. Source: USPTO, www.uspto.gov: CNIPA, www.cnipa.gov.cn. **a**–**b** Counting type root canal files [85–87]. **c**–**g** Preventing stress concentration in root canal files [88–92]

Fractures of root canal files are classified into fatigue fractures due to the excessive use and stress concentration fractures due to structural problems. Li M and Zhang DB proposed root canal files [85–87] that could count the number of times they were used, as shown in Fig. 9(a, b). Manual counting and electronic counting are the two counting structures. A controlled method [88–90] was proposed in the pieces of reference for locating root canal files fractures caused by stress concentration. When excessive torque or repeated use causes metal fatigue, the files will break at their setting to prevent fracture in the root canal. The structure of this type of root canal file is shown in Fig. 9(c–e), respectively. It was proposed to add a “stress dispersion part” [91, 92] between the conventional files and the handle in the reference, as shown in Fig. 9(f–g). Table 3 summarizes the methods of the root canal files based on the structure.

**Table 3:** Overview of special-structure methods of root canal files

Code	Special-structure	Method	Performance	Characteristics
US20110081623 [77]	STF	Mesh structure	The realization of different shapes and sizes	IS, HA
CN208114663 [78]	STS	M- and inverted M-wave	Adaptation of root canals	Uniform cutting
CN110497163 [79] CN110693615 [80]	STS	Brushed metal	Composite embryo process	GCR
WO2018002951 [81]	STS	Flexible filament arrangement	Basket shape	IS
US20140045142 [82]	STS	SWW	Brush sweep	GCR
US20110212413 [83]	STS	SWW and sandblasted	Sliding between files body and handle	IS
US9585731 [84]	STS	SWW with metal particles	With polishing function	GCR, HCE
CN209107620 [85]	STH	Counter	Simple counting	Easy to observe the number of uses
CN108542512 [86] CN108433831 [87]	STH	Counter	Visual counting	Direct observation of the number of uses
CN202437374 [88] CN209004247 [89]	STF	Setting recess	Ring grooves	IS, RSC
CN113081324 [90]	STF	SRS	Ring grooves	IS, RSC
EP3597141 [91]	STF	SRS	Spiral springs	IS, RSC
US20160128800 [92]	STF	SRS	Horizontal penetration port	IS, RSC

Special-structure/Method: *STF* Structure of the files, *STS* Structure of surface, *STH* Structure of Handle, *SRS* Stress relief section, *SWW* Spiral wound wire

Performance/Characteristics: *IS* Improve safety, *HA* Highly adaptable, *GCR* Good chip removal, *HCE* High cutting efficiency, *RSC* Reduction of stress concentrations

#### Special-section

Due to the symmetrical cross-section of the file body, conventional root canal files are inflexible and have difficulty following the tendency of the root canal to enter the tip. A fracture is likely to happen when the root canal is severely flexed. The asymmetric cross-section, or eccentric section, is a newly proposed design. It has a serpentine wave-like motion and points in contact with the root canal wall. The asymmetric cross-sectional design gives the root canal files more space to accommodate debris, facilitating debris removal, and its serpentine motion makes it easier to access the root tip. It has also been found that the eccentric cross-sectional design reduces the torque exerted on the instruments, which improves their preparation efficiency and facilitates root canal formation when preparing narrow curved root canals. An important aspect of the fatigue resistance of root canal files is their cross-sectional design, the continuous improvement of which will optimize fatigue resistance.

**Fig. 10:**
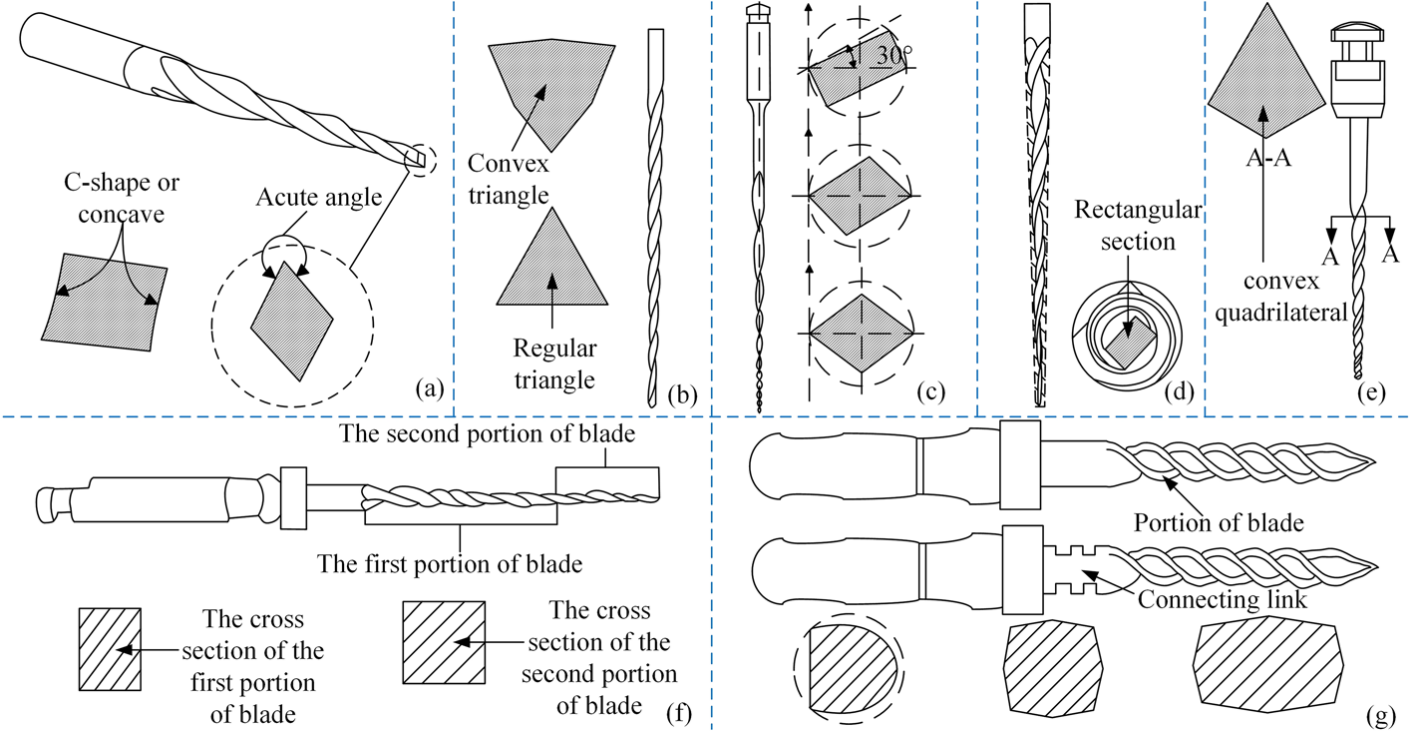
Special-section root canal files. Reproduced with permission. Source: USPTO, www.uspto.gov: CNIPA, www.cnipa.gov.cn. **a** A parallelogram cross-section [93]. **b** A convex triangular design [94]. **c** A non-isometric cross-section [95]. **d** A rectangle-shaped [96]. **e** A convex quadrilateral [97]. **f** A rectangular cross-section and a square cross-section [98]. **g** A quadrilateral cross-section [99]

A parallelogram cross-section [93] was proposed by Shotton V, which has an acute angle and an asymmetric rotation axis. Its center of mass is not on the axis of rotation, which can produce a larger scraping range. Figure 10(a) shows the schematic diagram of the file's structure with the cross-section of the body at different positions. A convex triangular design was proposed by Long XP to improve the fatigue strength of root canal files in the circumferential and axial directions [94]. Figure 10(b) shows a schematic diagram of the structure of the files. Wang Z proposed root canal files with a non-isometric cross-section [95]. As shown in Fig. 10(c). Due to the difference in axial dimensions, the bending deformation capacity is poor in the direction of the long axis of the cross-section. However, it has a good deformation ability in the short-axis direction. Thus, flexibility is improved while strength is maintained. Zhong S used integral machining and molding, and the cross-sectional shape [96] and the center of rotation of the file bodies were designed eccentrically. Figure 10(d) shows a cross-section of the files with one of the implementation forms. A rectangle-shaped file cross-section only has two adjacent corners on the cutting boundary when it is designed as a rectangle. The force on the root canal files is greatly reduced. Yue B proposed that the cross-section [97] is a convex quadrilateral, where three angles are obtuse or right angles and the other angle is acute. As in Fig. 10(e), the files allow overcutting of the dentin. Liu S proposed root canal files with a double-edged section [98], where the first edge was machined from a cone with a rectangular cross-section and the second edge was machined from a cone with a square cross-section. Figure 10(f) shows a schematic diagram of the shape of the files with enhanced chip evacuation. Liu S then proposed that the files have a quadrilateral cross-section [99–101], the convex ribs project outward and the edge part is twisted by the cone. Using this method, the root canal files automatically conform to the root canal shape, which facilitates its entry and prevents the occurrence of lateral penetration. Figure 10(g) depicts the structure of the files and the cross-section of the edge.

**Fig. 11:**
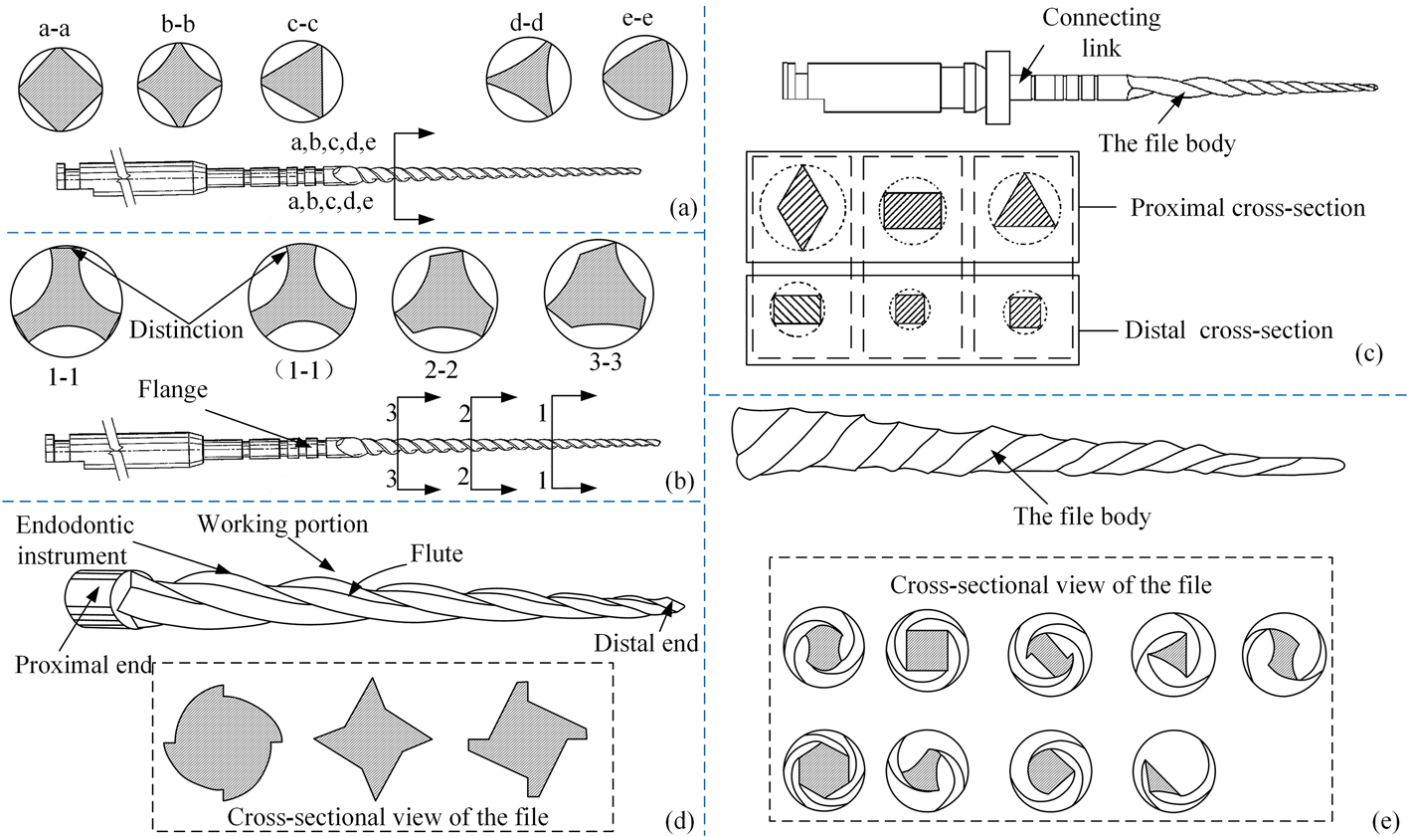
Special-section root canal files. Reproduced with permission. Source: USPTO, www.uspto.gov: CNIPA, www.cnipa.gov.cn. **a** The cross-sectional shape of the root canal files in the reference [102]. **b** Schematic diagram of the cross-section of the root canal files [103] in different positions. **c** Proximal cross-sections and distal cross-sections in the reference [104]. **d** Cross-sectional view of the files in the reference [105]. **e** Cross-sectional view of the files in the reference [106]

Besides the cases described above, there are also ways to set different cross-sectional shapes according to different needs. Figure 11(a) illustrates the cross-sectional shape [102] of the root canal files in the reference, which ensures flexibility and strength. A concave helical groove [103] with continuous intervals was proposed by Jaunberzins A for the handle section. Additionally, it increases flexibility, reduces torsional resistance, and extends the prepared length. Figure 11(b) shows a schematic diagram of the structure of the files. Zhou L proposed that the cross-section [104] of the files gets smaller as it gets closer to the distal end. There is a difference in cross-sectional shape between the distal and proximal ends of the files. And in any two sections, the ratio of the area of the section near the proximal end to the area of its outer circle is not greater than the ratio of the area of the section near the far end to the area of its outer circle. As shown in Fig. 11(c), the strength of the middle section of the root canal files and the toughness of the distal end is ensured. William B proposed a polygon cross-section [105] at the proximal end of the files, which gradually becomes a square at the distal end. The cut angle of the files is achieved by rotating it in a positive direction, whereas the scraping angle is achieved by turning it in a negative direction. Figure 11(d) shows a schematic cross-sectional view of the files and their various embodiments. McSpadden JT proposed multi-tapered root canal files [106]. There are at least two grooves on the body of the file, which are, respectively, thinned along with the root canal files according to a predetermined taper function to form different sections. Torque loading should be reduced and the tendency to screw into the canal should be reduced. Figure 11(e) shows a schematic diagram of the profiles of the files and partial transverse cross-section views of additional alternative embodiments of a multi-tapered endodontic instrument. This kind of file meets the different performance priorities of the proximal and distal ends by setting polygons of different shapes at the proximal and distal ends of the body of the file. Under the premise of ensuring cutting efficiency, the body of the file can take into account the chip removal ability and strength requirements. Table 4 summarizes the special-section shape and their illustrations.

**Table 4:** Overview of special-section methods of root canal files

Special-section shape	Method	Performance	Code	Illustrations
Parallelogram	Asymmetrical design	LCS, GCR	US20150216624 [93]	
Convex triangle	Adaptation to different patients	Circumferential HCE, axial FR	CN204446150 [94]	
Non-isometric section	Non-isometric section	GF	CN206044757 [95]	
Eccentric design	Asymmetrical design	Non-breakable, IS	CN206910405 [96]	
Convex quadrilateral	Can overcut	LCS	CN209884369 [97]	
Rectangular	Includes two cutting edges	GCR, IS	CN206995351 [98]	
Quadrilateral Convexity	With convex ribs	HA, GF	CN204446152 [99] CN204446154 [100] CN204446155 [101]	
Irregular shape	Inner joint round	FR, IS	US20170135786 [102]	
Irregular shape	Continuous interrupted concave spiral grooves	Variable and flexible length, LCS	US20100297578 [103]	
Gradual reduction	Different section shapes	HA, GF, LCS	CN209611357 [104]	
Concave polygon	Different rotation directions correspond to different functions	GV, HCE, IS	US20100040994 [105]	
Multi-conical	According to different taper functions	GCR, HCE	US20100255442 [106]	

*LCS* Large cleaning space, *GCR* Good chip removal, *HCE* High cutting efficiency, *FR* Fatigue resistance, *GF* Good flexibility, *IS* Improve safety, *HA* Highly adaptable, *GV* Good versatility

### Additional function

Clinicians and patients look for safety and cleaning ability in root canal files when they use them in clinical settings. However, even with root canal files, problems, such as instrument separation, accidental falling off, secondary infection of the dental pulp, inadequate preparation, or over-preparation, may still occur. Root canal files perform insufficiently, which is obvious from their single function. Insufficiently performing root canal files cause these problems. The methods of adding additional functions have been proposed by scholars as a means of solving these problems. The paper categorizes them into three types: anti-fall methods, anti-infection methods, and anti-error methods.

#### Anti-fall

Clinically, root canal files are used in a humid oral environment, which is susceptible to accidental falls. Two types of accidental falls of root canal files are caused by saliva lubrication and loose connections between the file’s body and handle. When used in clinical settings, the root canal files are very close to the respiratory tract, throat, and other tissues. Accidental falling off will easily cause medical accidents. Recently, several methods have been developed to prevent falling off.

**Fig. 12:**
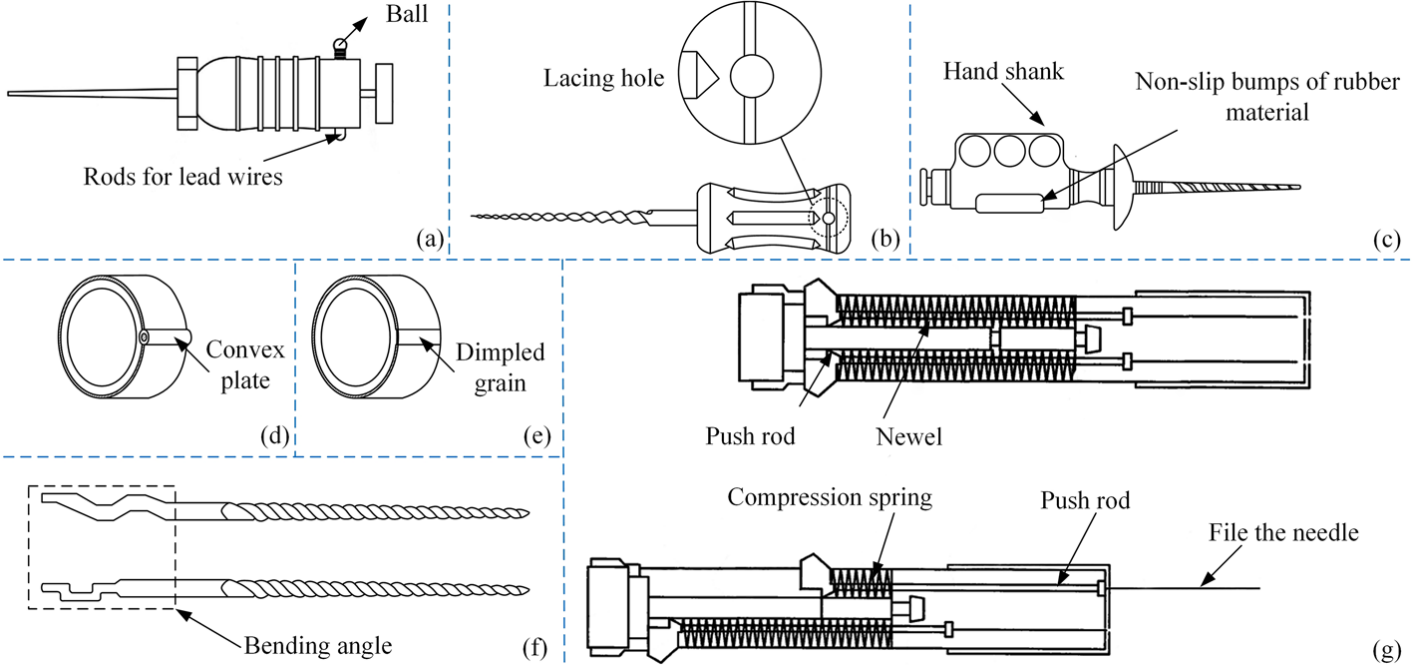
Anti-fall root canal files. Reproduced with permission. Source: USPTO, www.uspto.gov: CNIPA, www.cnipa.gov.cn. **a**–**b** The files with a rope threading hole [107, 108]. **c** Root canal files with a stable handle [110]. **d**–**e** Magnetic bracelets prevent falling off [109]. **f** A bending structure prevents falling off [111]. **g** With a special cylindrical structure [112]

Hao ZY and Chi HY proposed files with a rope threading hole [107, 108], as shown in Fig. 12(a, b). Luo WC proposed a file equipped with a magnetic bracelet [109]. Figure 12(d, e) shows the structure of the magnetic bracelet. To prevent falling, the handle of the root canal files can work with the magnetic bracelet, but the direction of the resultant force of magnetic force and external force is somewhat difficult to control. A root canal file with a handheld part was proposed by Wang LX to solve the issue of poor holding stability [110]. The structure is shown in Fig. 12(c). But it is bulky and inconvenient to hold.

As shown in Fig. 12(f), Sun QX proposed a bending structure [111] for the loose part of the body of the file and handle. This could solve the problem of sliding between the root canal files and handle. Zheng YX proposed the files with the structure [112] shown in Fig. 12(g). The end of the body of the file is provided with a cylindrical body, which is provided with some push rods for fixing the file's needle, and the push rods are, respectively, sleeved with a compression spring. The structure can select files of different specifications according to needs and can also adjust the working length of the files. The special cylindrical structure can also prevent the connecting part between the file's body and the handle from loosening. In Fig. 12(g), the left side is the schematic diagram of the root canal files pressing out the body of the file.

#### Anti-infection

During root canal therapy, pulp infections are common. Dentin fragments and root canal files that are accidentally broken in the root canal can cause pulp infection. At present, there are solutions, such as combining ultrasonic technology [96] or disinfection light source [101] with traditional mechanical root canal files, combining chemical flushing with mechanical preparation [97–100], and special tip structure [103, 104] for broken files. It is no longer simply a matter of cleaning up the necrotic pulp in a root canal, but the process of preparing a root canal is becoming simpler and safer.

**Fig. 13:**
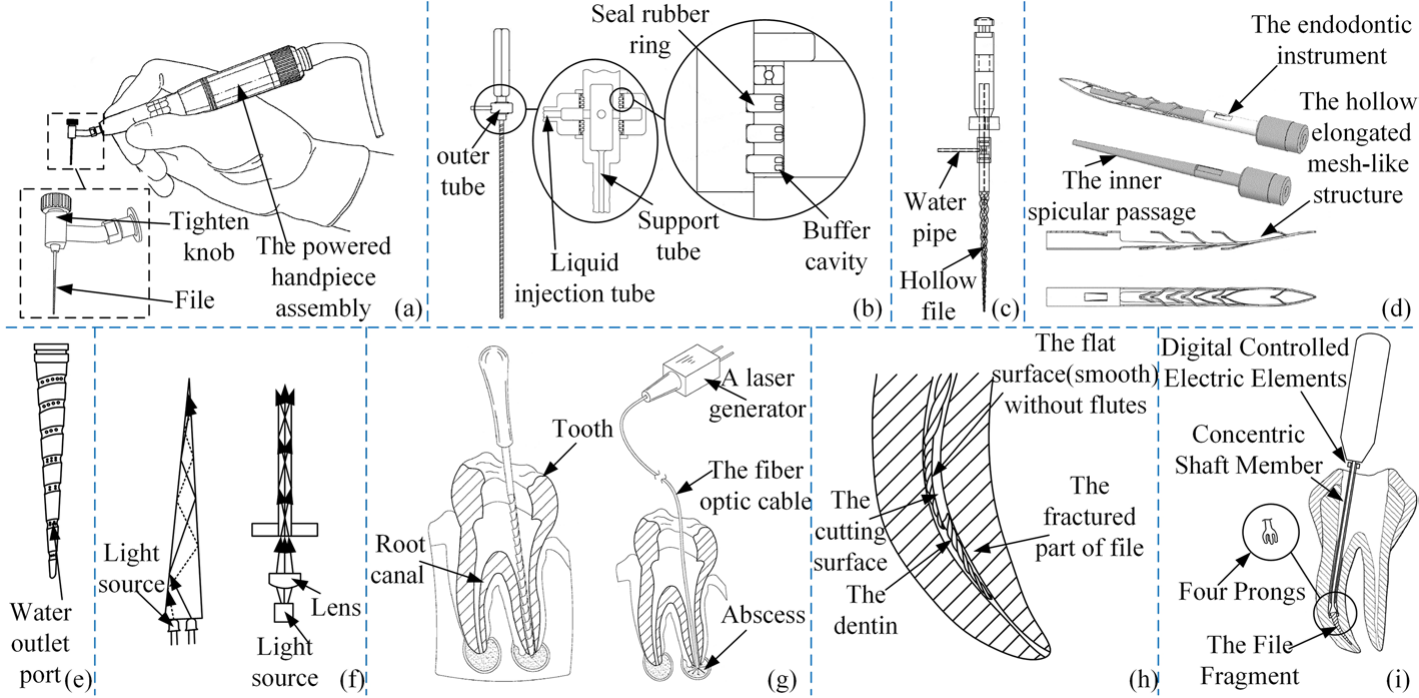
Anti-infection root canal files. Reproduced with permission. Source: USPTO, www.uspto.gov: CNIPA, www.cnipa.gov.cn. **a** Root canal files with ultrasound function [113]. **b**–**e** Root canal files with flushing function [114–117]. **f** Root canal files with light anti-inflammatory function [118]. **g** Root canal files with laser anti-inflammatory function [119]. **h** Can bypass fractured root canal files [120]. **i** Can clamp fractured root canal files [121]

Ramos CAS combined ultrasonic technology [113] with root canal files to help destroy necrotic tissue due to the difficulty of removing debris, the structure of which is illustrated in Fig. 13(a). As shown in Fig. 13(b–e), scholars proposed applying the flushing function [114–117] to the root canal files to remove debris. Sterilization and disinfection follow the cleaning of the dentin. To better restore the prepared root canal, light-based root canal files were proposed in the reference [118]. It can emit light of one or more wavelengths, which can realize a variety of therapeutic benefits, for example, disinfection, tissue regeneration, reconstruction of vascular tissue, and reduction of inflammation or pain. Figure 13(f) shows a schematic diagram of two embodiments of the files. A plurality of files [119] is provided and each file is insertable into a root canal. The fiber optic cable is insertable into the root canal to communicate the laser light onto an abscess for eliminating the abscess without conventional surgical intervention, as shown in Fig. 13(g).

For accidental fracture of stuck files fragments. Radwan S proposed files [120] that can bypass the fractured part. As shown in Fig. 13(h). Later, he proposed a champing-type [121] root canal file that can remove the fractured files. These root canal files can help complete a complete root canal therapy without the influence of fractured files, as shown in Fig. 13(i)

#### Anti-error

The most common cause of pulp failure in root canal therapy is insufficient or excessive preparation of the root canals. Often, these complications occur as a result of a poor understanding of root canal length before the operation, so it is especially important to measure root canal length accurately. An apical locator is a traditional tool for measuring the working length of the root canal. During operation, large errors are often caused by cursor movement or personnel errors. Measurement steps are not only cumbersome but also inaccurate and time consuming. In addition, estimating the length of a root canal may lead to over-preparation or under-preparation due to mistake operation. Several methods to prevent error preparation have been proposed in recent years in light of these problems.

**Fig. 14:**
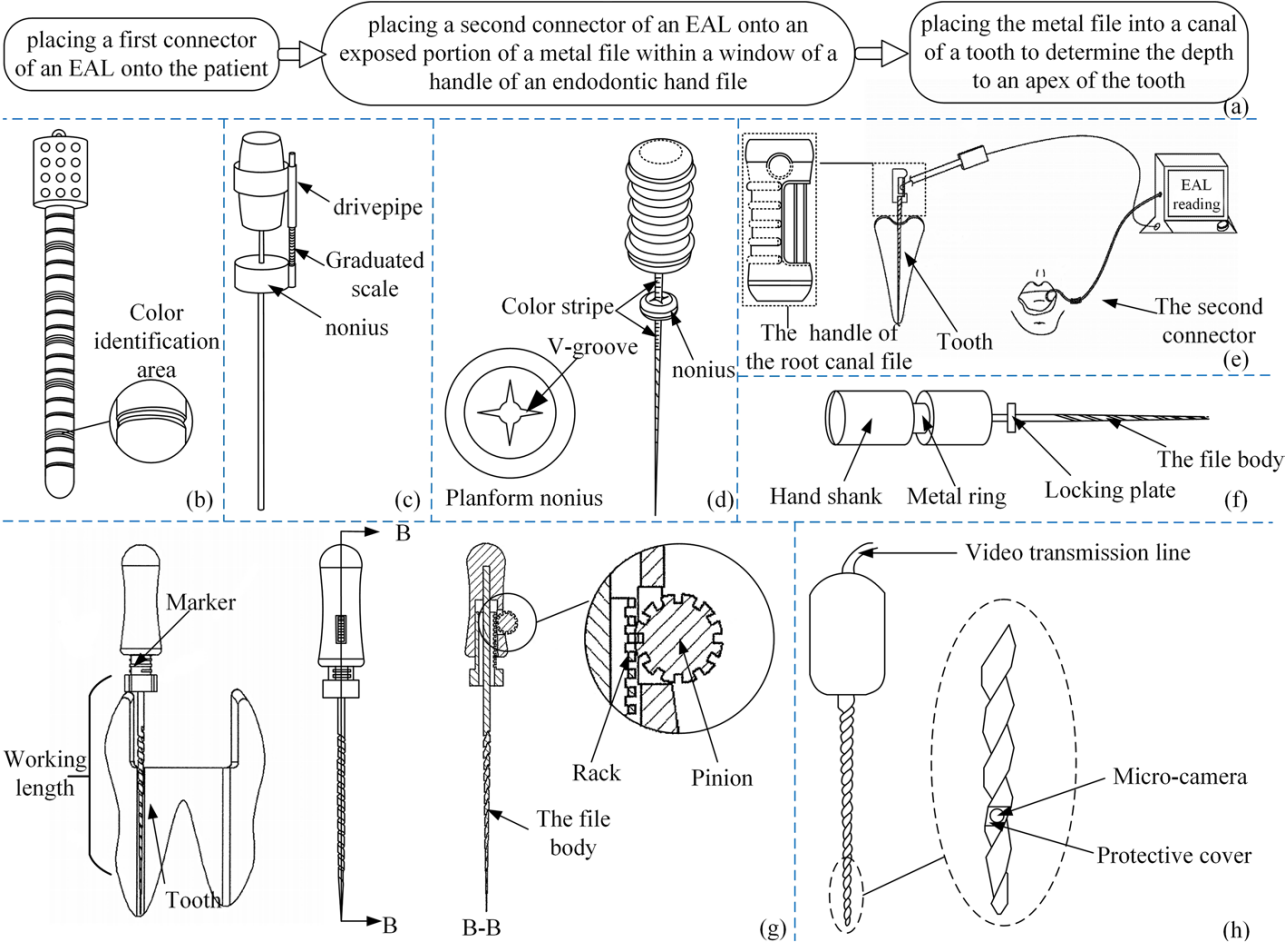
Effective preparation of root canal files. Reproduced with permission. Source: USPTO, www.uspto.gov: CNIPA, www.cnipa.gov.cn. **a** Process of using the electronic apical locator. **B**–**d** Root canal files for measuring root canal length [122–124]. **e** Root canal files with exposed window [125, 126]. **f** Structure to prevent disturbance [127]. **g** Structures limiting the working length of root canal files [128]. **h** Microscope working schematic [129]

The references [122–124] provided root canal files for measuring root canal length, and Fig. 14(b–d) illustrates its structure. By setting an exposure window at the handle of the root canal files, Bagheri MJ attempted to overcome the problem of the traditional electronic apical locator sliding [125] when attached to the body of the files. The window structure is shown in Fig. 14(e). The snap of the electronic apical locator can be connected to the metal handle in the window exposure. These steps in Fig. 14(a) are performed in the root canal therapy of a patient. A solution [126] proposed by Du Y is to integrate the aluminum wire of the electronic apical locator with the metal of the handle so that the process of measuring root canal length does not involve repeated clamping and removal. Additionally, the measuring instrument collet can be avoided from affecting the field of vision and operation. Cai proposed a metal ring [127] fixed to the handle to solve the interference problem between the clip and the stopper. Figure 14(f) shows the structural diagram of the files. Curry AD proposed a method to limit the length of root canal files into root canal [128]: Root canal files have an adjustment scale and a nonius on the body. Once inserted into the root canal, the washer should be fixed to the tooth surface, which is shown in Fig. 14(g). Zhang XR proposed setting a micro camera at the end of the files away from the handle and embedding a video transmission line in the files [129], as shown in Fig. 14(h). Through the transmission line, images captured in real time in the root canal can be transmitted to the external display device, so that you can observe the root canal in real time. The relevant files and their improved methods are summarized in Table 5.

**Table 5:** Overview of additional function methods of root canal files

Additional function	Method	Performance	Advantage	Code
Anti-fall	Stringing holes	Can be taken out by a string	IS	CN202477881 [107] CN214414938 [108]
	Magnetic bracelet	Can magnetically absorb files	IS	CN204890207 [109]
	Non-slip bumps	Increased friction with fingers	GC, GS	CN209899615 [110]
	Bent section	Increased stability with the handle	GS	CN203790060 [111]
	Cartridge	Addition of different root canal files	GC, GV	CN203564347 [112]
Anti-infection	Ultrasound technology	Activate chemicals	GCR	US20160022377 [113]
	Support tubes	Facilitate fluid injection	GCR	CN212089798 [114]
	Hollow files body	irrigate while treating	GCR	CN211796958 [115]
	Flushing equipment	Hollow files and inner specular passage	GCR	WO2021144465 [116]
	Several holes in the outer surface	Clean and transport dirt	GCR	CN208989188 [117]
	Sterilization unit	Reduce inflammation or pain	HTP	WO2018009864 [118]
	Laser generator	Eliminates the abscess	HTP	US20210244499 [119]
	Flat surface	Can bypass fractured files	IS, GF	WO2021186224 [120]
	Prongs	Can remove the fractured files	IS, GC	US10813719 [121]
Anti-error	MD	Different colored marking zones	IS	CN207545234 [122]
	MD	Nonius	IS	CN206403877 [123]
	MD	Improved nonius	IS, GS	CN205279948 [124]
	Exposure Window	Connection to electronic apical locator	AM, GC	US20190125508 [125]
	Setting up a one-piece structure	Connect the metal wire to the handle	AM, GC	CN206381250 [126]
	Metal rings	Fixed connection to files body	GS, GC	CN206151607 [127]
	Nonius	Nonius can be fixed with different lengths	IS, GS	US20110300506 [128]
	Miniature camera	Observation of the inside of the root canal	IS, GVI	CN205626145 [129]

*MD* Measuring device, *IS* Improve safety, *GS* Good stability, *GV* Good versatility, *GCR* Good chip removal, *HTP* Has therapeutic properties, *GF* Good flexibility, *GC* Good convenience, *GS* Good stability, *GVI* Good visibility, *AM* Accuracy measurement

## Discussion

**Fig. 15:**
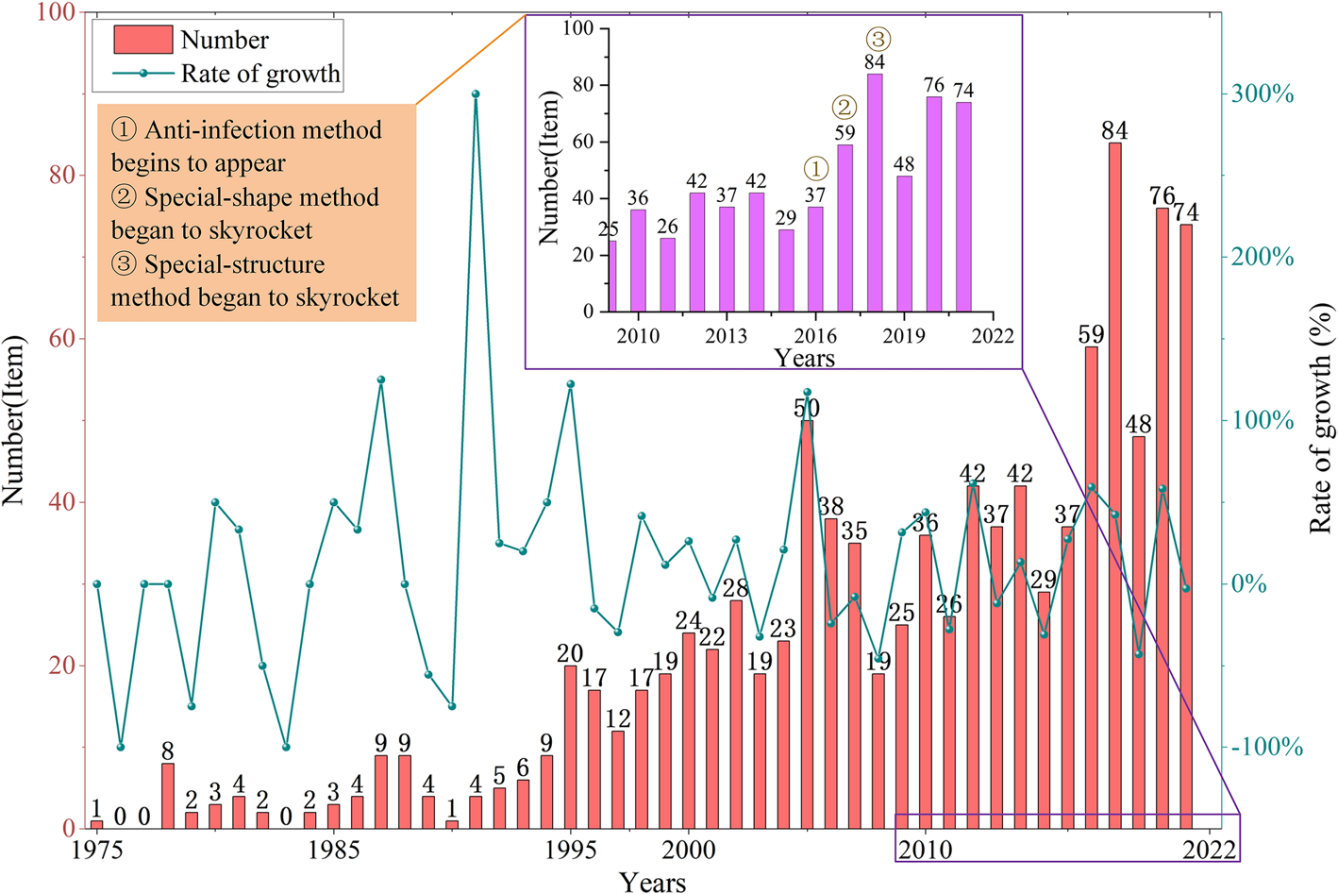
The growing trend of root canal files

As shown in Fig. 15, we can see that the number of patents for root canal files has increased each year since the first patent for the root canal file, US04028810, was filed in 1975. This proves that in recent years there has still been a lot of researches into how to improve the performance of root canal files. By reviewing the paper, the development of root canal files in the last decade has been dominated by geometric designs. The geometric design [130, 131] influences the movement of the root canal files and determines the forces applied to files in the root canal. Further, due to the advantages of composite structures, such as hollow structures, compressibility, the ability to mount specific flushing devices, and continuous chemical preparation alongside mechanical preparation, research hotspots have shifted from the cross-section of root canal files to open composite structures. However, there are some limitations to the preparation of special root canals. Therefore, the application of the correct instrumentation in combination with the shape of the root canal can improve the shaping and cleaning ability [132] and reduce complications. Figure 15 shows when anti-infection methods began to appear in large numbers, indicating that patients are taking the safety of root canal treatment more seriously. In the future, anti-infection methods will be a hot new research topic.

However, as the number of ways to improve the performance of root canal files has increased, the performance of root canal files has become more and more sophisticated. Several studies have therefore investigated the differences from several perspectives [133], finding differences in design, phase transformation temperatures, and mechanical behavior of instruments. The low-cyclic fatigue resistance of counterfeit instruments makes them unsafe systems. To compare the performance of different root canal files in vivo and in vitro experimental studies, multimethod assessments [134] can be considered one of the main advantages of current research. This methodological approach allows for a more comprehensive assessment regarding the properties of the tested instruments, as it avoids “knowledge compartmentalization” a phenomenon in which knowledge structures about a specific domain are composed of several separate parts [131]. Understanding these characteristics may help clinicians make decisions regarding which instrument to choose for a particular clinical situation.

Although we have now improved the performance of root canal files, there are more factors affecting root canal therapy than just the performance of the files. As mentioned above, the experience of the clinician and the complexity of the root canal affect the success rate. The same root canal file used by different clinicians may give different results for root canal therapy. It may be that experienced clinicians have a better understanding of what kind of root canal file and what form of motion (reciprocation or OTR) is appropriate for the root canal. Root canal therapy capacity, working length variation, mid-axis offset, bending variation, root canal therapy time, and success rate all vary with different file motions. It is important to study the ability of root canal therapy and shaping with different file motions (reciprocation or OTR). Reciprocating motions can reduce the formation of dentin cracks to some extent in terms of safety compared to conventional rotary motions [135–137], but this advantage needs to be based on the selection of a suitable model according to the size of the root canal. Whereas the introduction of apical debris is closely related to both the motion pattern and the cross-sectional design of the instrument [138–140], debris removal is facilitated if a large amount of flushing fluid is used during root canal therapy [141]. Reciprocating Nitinol files are made from especially tensile machined and heat-treated M-wires, which are significantly more resistant to cyclic fatigue behavior and wear than other Nitinol instruments [142, 143]. In terms of microbial clearance, when applying reciprocating motion for root canal therapy, although the mechanical preparation time is significantly reduced, the flushing time should be longer than when using a continuous rotary motion system for root canal therapy due to the need to flush with an adequate amount of flushing fluid [144]. The reciprocating motion has the least change in working length and root canal curvature in terms of natural root canal morphology maintenance [145]. The two main directions affecting working length variation are the curvature of the root canal and the adjustment of the coronal access [146], and the more curved the root canal, the more pronounced the straightening effect of the adjustment of the coronal access. In terms of efficiency, the application of a single file reciprocating motion root canal therapy system avoids frequent instrument changes and, together with its greater dentin-cutting capacity, improves the efficiency of root canal therapy [147, 148]. And the OTR motion, Optimum Torque Reversal, is a torque sensitive, round-trip motion mode. OTR motion contributes to the release of spin-in forces compared to conventional rotational motion, and continuous rotation generated notably higher peak torque values than OTR motion [149]. The OTR movement reduces the torque and wedge forces during the crown-down preparation phase of the crown-down method and the apical preparation phase of the single-length method. The number of instrument separations can therefore be reduced.

**Fig. 16:**
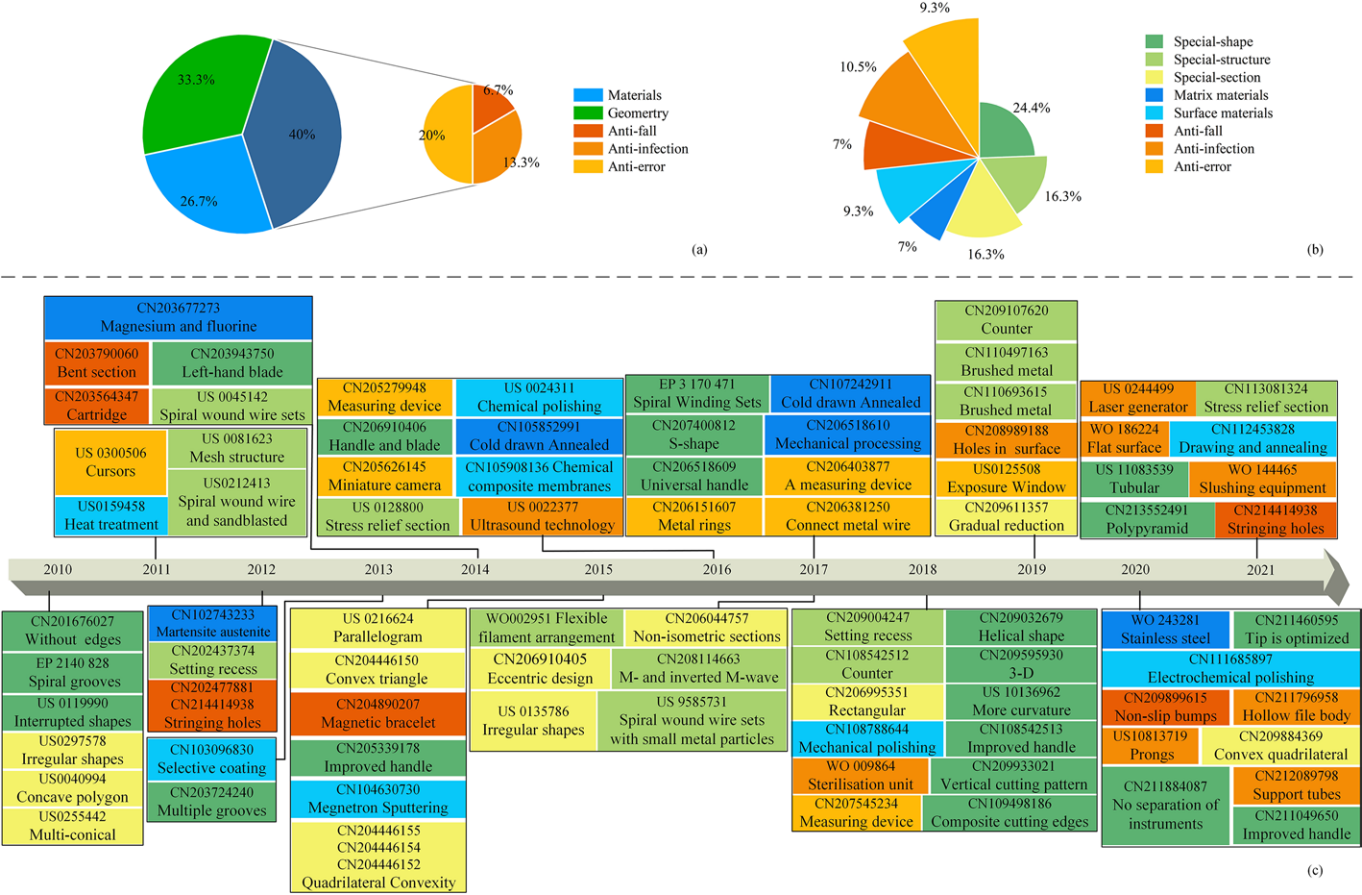
The patents of this paper. **a–b** Improvement method classification percentage. **c** Patent numbers of root canal files appearing in the paper

This paper reviewed improved methods of root canal files. Figure 16(a, b) shows the percentage of root canal patents that is classified by the improved methods. According to the patents selected for this paper, the additional function methods account for the largest proportion of the three major classifications of root canal archives, at 40%. From Fig. 16(b), it can be found that among the eight subcategories of improvement methods, the special-shape methods accounted for the highest percentage at 24.4%, while the anti-fall methods and the matrix material methods accounted for the lowest percentage at 7.0%. The patents appearing in the paper are ordered in Fig. 16(c) by improved methods and time of appearance. The specific improvements to each root canal file are also shown in Fig. 16(c). The key techniques and features are represented in Table 6.

**Table 6:** Overview of three improved methods of root canal files

Improvement type	Method	Key technology	Advantage	Limitation
Material	Matrix	Ultrafine crystal Heat treatment 3D printing	Flexibility Anti-fatigue	Tedious process steps High manufacturing cost
	Surface	Polishing Magnetron sputtering process Coating Technology	Corrosion resistance Inhibition of ion precipitation	Unstable surface coating Low utilization of target material
Geometry	Special-shape	Improvement of internal force/external force Geometric theory	Large preparation space Adaptation of root canal alignment	Inadequate cleaning strength Unstable files structure
	Special-structure	Composite structure theory Stress theory	Easier to carry the chemical irrigation agent into the root canal Prevention of stress concentration	Debris tends to get stuck in the root canal files Weak chip removal function
	Special-section	Full use of shape and structure information Eccentric section design theory	Large chip space Easier access to the root apex	Manufacturing complexity Eccentric structure is easy to lose control
Function	Anti-fall	Structural Design Composite principle	Good grip stability Easy replacement of root canal files	Bulky and heavy Easily stained with dirt
	Anti-infection	Ultrasound Technology Optical therapy technology	Easy to clean dentin Treatment of root canals and endodontics	Expensive to manufacture Difficult to operate
	Anti-error	Use of special structures Micro Photography	No apical perforation Security Ability to measure root canal length	Cannot precisely determine root canal depth The parts are small and inconvenient to operate

### Future trends

#### Material

**Fig. 17:**
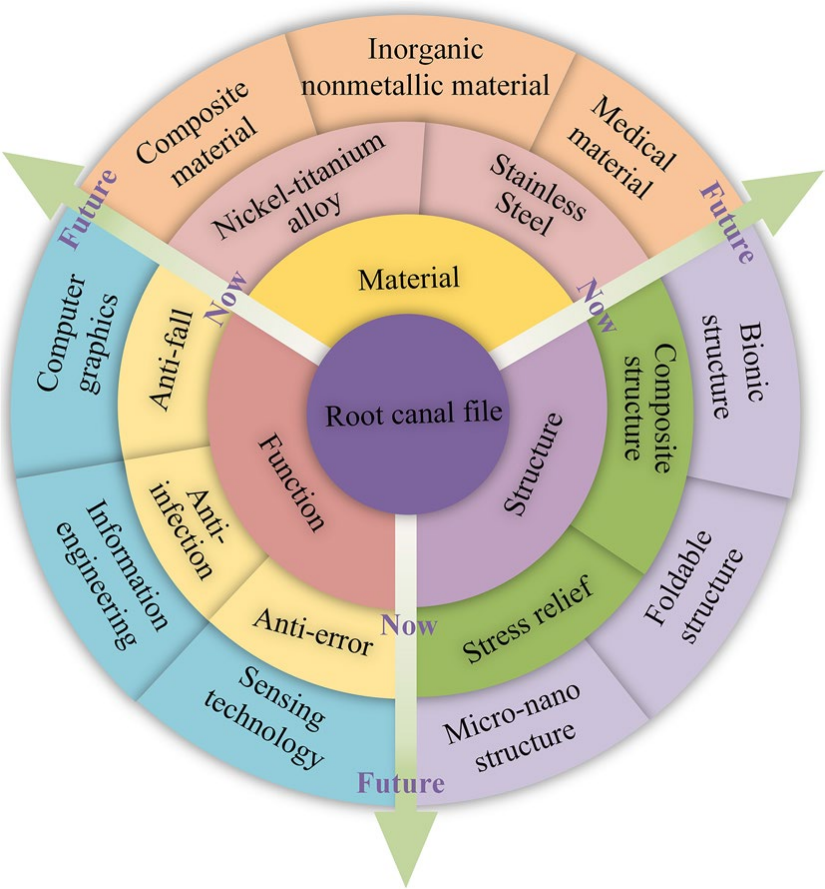
Future directions of root canal files and future root canal therapy procedures

The methods for improving the performance of root canal files proposed in this paper pose several problems. Materials for root canal files should be developed to solve the problems encountered in the clinical use of root canal files. Considering the limitations of the material, it is possible to study experimentally how to instrument fatigue fracture that occurs during root canal therapy. According to the research results, find alternative materials, such as graphene [150]. Because graphene shows good toughness and high flexural strength, it can adsorb and desorption various atoms and molecules, and has biocompatibility and stability. As part of the root canal therapy process, it can serve as a cleaning and sterilization agent. By using graphene as a base material or surface treatment material, i.e., a highly thermally conductive graphite film, the mechanical properties of the root canal file will be greatly improved. Alternatively, inorganic non-metallic materials are used as the matrix materials for root canal files, such as ceramic materials [151], or medical materials, such as medical-grade Nitinol powder [152]. Vigorous development of medical additive manufacturing technology, breakthrough medical grade titanium powder and nickel-titanium alloy powder and other key raw material constraints, can be a new idea in the manufacture of root canal files. Three-dimensional (3D) printing technology [153] is driving changes in the medical and health care industry, and titanium and titanium alloys [154], as biomedical materials with excellent performance are developing at an alarming rate. The combination of the two will help push personalized medicine to shine. Or making composite materials [155], such as preparing antibacterial materials by polymer polymerization, surface functionalization, and derivatization, and coating biomimetic micro nanostructures with bactericidal function. To prevent instrument separation, change the surface morphology and structure through physical methods. These future directions of materials are shown in Fig. 17.

#### Structure

The mechanical properties are influenced by the structure of the root canal file. Three future trends in the structure were discussed in this section. Bionic structures [156, 157] are one of them. As bionic technology keeps evolving, through the design morphology under the biological incentive mode and integration of multi-disciplinary knowledge to learn, simulate, and copy the function, behavior, or structure of organisms, to develop the root canal files with bionic structure. These methods can improve the debris removal ability and cutting effect of root canal files. For example, the tongue of the pangolin [158] can clean out anthills quickly and accurately without causing harm to the anthills, and its method of cleaning out anthills is similar to that of root canal therapy and is worth learning from. The second is the foldable structure [159]. It has a unique space-occupying volume. To introduce a foldable structure that can remove the necrotic pulp without over-preparing the root canal, thereby ensuring the maximum retention of the affected teeth. Finally, we can also start with the size of the structure and introduce a micro nanostructure [160]. Root canal files can be converted into micro nanorobots to realize minimally invasive treatment, remove the necrotic dental pulp, and eliminate bacteria. After you input the digital root canal therapy information, the robot will select the most suitable surgical scheme in the database for this kind of root canal. Automated preparation can replace manual operation completely. These structures are shown in Fig. 17.

#### Function

In the future, more functions will be added to root canal files to simplify the treatment. Given the current problems encountered with the clinical use of root canal files, this section proposed the following aspects of future functions. For example, the micro-display will be created inside the root canal, by using 3D display technology [161], and the clinicians can observe the progress of root canal therapy in real time to determine the next step needed. With sensing technology [162] and computer graphics technology [163], it is possible to perform under-preparation detection, over-preparation detection, collision detection, and detection of whether the root tip is reached, allowing for safer and more complete preparations. As computer and artificial intelligence technologies [164] continue to develop, a large number of root canal therapy cases have been compiled on the Internet, which can provide more comprehensive information on the problems of root canal therapy. So, before formal root canal therapy, the root canal model can be generated based on the patient's real dentition. Clinicians can then select the appropriate root canal files based on the patient's 3D root canal model with biomechanical properties and perform root canal therapy. When faced with this type of root canal, the large number of root canal models will help clinicians choose the right root canal files. A trial preparation with root canal files will be conducted based on the virtual root canal model. The sensor on the root canal files will examine the problems encountered. If the files are not suitable for preparing that type of root canal at this time, the clinician can replace them in advance without causing a break during clinical use. It is possible to anticipate the risk and make timely adjustments. In Fig. 17 you can see some general directions based on the additional functional methods.

## Conclusion

In this paper, we analyze the problems encountered in the use of root canal files and the factors affecting their performance. Existing improved methods (theoretical research) of root canal files have been surveyed and classified into three categories, i.e., material-based methods, geometry-based methods, and those based on additional function methods. The basic information of each classification and the advantages and limitations of each are also described in detail. And this paper further explains the percentage of different methods and determines the development trend of root canal files at this stage and the popular advance direction. In addition, this paper proposes the future development direction for root canal files based on three principal methods. The future progress of root canal files will be guided. This paper understands the state of the art and identifies future research directions for the improved methods of root canal files. It contributes to the accuracy, effectiveness, and reliability of root canal therapy. In addition, the proposal and overview of the improved methods for root canal files are of great importance in promoting the precise diagnosis and treatment of dental pulp disease and periapical disease.

## Data Availability

Not applicable.

## Abbreviations

NiTi: Nickel-titanium
MA: Martensite
AU: Austenite
CDA: Cold drawn annealed
HT: Heat treatment
CP: Chemical polishing
GF: Good flexibility
FR: Fatigue resistance
WR: Wear resistance
IS: Improve safety
HCE: High cutting efficiency
GS: Good stability
GCR: Good chip removal
SS: Space spiral
LR: Low resistance
GC: Good convenience
NCE: No continuous embedding
IS: Improve safety
GV: Good versatility
GVI: Good visibility
STF: Structure of the file
STS: Structure of surface
STH: Structure of handle
SRS: Stress relief section
SWW: Spiral wound wire
IS: Improve safety
HA: Highly adaptable
GCR: Good chip removal
HCE: High cutting efficiency
RSC: Reduction of stress concentrations
LCS: Large cleaning space
GCR: Good chip removal
HCE: High cutting efficiency
FR: Fatigue resistance
GF: Good flexibility
IS: Improve safety
HA: Highly adaptable
GV: Good versatility
MD: Measuring device
IS: Improve safety
GS: Good stability
GV: Good versatility
GCR: Good chip removal
HTTP: Has therapeutic properties
GF: Good flexibility
GC: Good convenience
GS: Good stability
GVI: Good visibility
AM: Accuracy measurement
OTR: Optimum torque reversal
3D: Three-dimensional
